# Gene regulatory network reveals oxidative stress as the underlying molecular mechanism of type 2 diabetes and hypertension

**DOI:** 10.1186/1755-8794-3-45

**Published:** 2010-10-13

**Authors:** Jesmin, Mahbubur SM Rashid, Hasan Jamil, Raquel Hontecillas, Josep Bassaganya-Riera

**Affiliations:** 1 Department of Genetic Engineering and Biotechnology, University of Dhaka, Dhaka-1000, Bangladesh; 2 Department of Computer Science, Wayne State University, Michigan, USA; 3 Nutritional Immunology and Molecular Nutrition Laboratory, Virginia Bioinformatics Institute, Virginia Polytechnic Institute and State University, Blacksburg, VA 24061, USA

## Abstract

**Background:**

The prevalence of diabetes is increasing worldwide. It has been long known that increased rates of inflammatory diseases, such as obesity (OBS), hypertension (HT) and cardiovascular diseases (CVD) are highly associated with type 2 diabetes (T2D). T2D and/or OBS can develop independently, due to genetic, behavioral or lifestyle-related variables but both lead to oxidative stress generation. The underlying mechanisms by which theses complications arise and manifest together remain poorly understood. Protein-protein interactions regulate nearly every living process. Availability of high-throughput genomic data has enabled unprecedented views of gene and protein co-expression, co-regulations and interactions in cellular systems.

**Methods:**

The present work, applied a systems biology approach to develop gene interaction network models, comprised of high throughput genomic and PPI data for T2D. The genes differentially regulated through T2D were 'mined' and their 'wirings' were studied to get a more complete understanding of the overall gene network topology and their role in disease progression.

**Results:**

By analyzing the genes related to T2D, HT and OBS, a highly regulated gene-disease integrated network model has been developed that provides useful functional linkages among groups of genes and thus addressing how different inflammatory diseases are connected and propagated at genetic level. Based on the investigations around the 'hubs' that provided more meaningful insights about the cross-talk within gene-disease networks in terms of disease phenotype association with oxidative stress and inflammation, a hypothetical co-regulation disease mechanism model been proposed. The results from this study revealed that the oxidative stress mediated regulation cascade is the common mechanistic link among the pathogenesis of T2D, HT and other inflammatory diseases such as OBS.

**Conclusion:**

The findings provide a novel comprehensive approach for understanding the pathogenesis of various co-associated chronic inflammatory diseases by combining the power of pathway analysis with gene regulatory network evaluation.

## Background

Type 2 diabetes (T2D) is a chronic disorder of carbohydrate, fat and protein metabolism. The prevalence of diabetes is increasing worldwide. According to the recent World Health Organization (WHO) estimates, more than 180 million people worldwide have diabetes. This number is likely to be more than double by 2030. There will be 300 million people with diabetes by the year 2025 [1]. It is estimated that the developing countries will bear the brunt of diabetes epidemics in the 21st century [1,2]. Mounting evidence indicates that obesity along with increased rates of other chronic inflammatory diseases, such as hypertension (HT) and cardiovascular diseases (CVD), are highly associated with T2D [3]. How all theses complications arise and manifest together is yet to be solved. Thus, understanding the underlying molecular mechanisms of T2D is essential for developing more targeted and effective therapies and preventive approaches against diabetes-related complications.

The integration of computational biology and "omics" science facilitates an understanding of the mechanisms of diseases in an integrative way, including elucidation of complex networks of genes and proteins along with their regulatory networks [4-7]. Protein-protein interactions (PPI) regulate nearly every living process. Availability of high-throughput genomic data has enabled unprecedented views of gene and protein co-expression, co-regulations and interactions in cellular systems. The approach of this present study was to build an integrated network model system comprised of high-throughput genomic data along with associated computational analysis that would enable predictions of systems level view of normal and aberrant genes, their connections and their functions- to elucidate the underlying molecular mechanisms. Herein we mined genes differentially regulated through T2D and their "wiring" which will provide a more complete understanding of the overall gene network topology and their roles in disease progression. By elucidating these networks, exploring how they have been linked and characterizing how they have evolved- novel disease markers can be identified. In this present study, by analyzing the genes related to T2D, HT and OBS, a highly regulated gene-disease integrated network model was developed. PPI sub-networks containing the hub genes involved in T2D-associated signaling pathways revealed that the oxidative stress mediated regulation cascade, is the link, associated with the mechanism of pathogenesis of type 2 diabetes, hypertension and other inflammatory diseases such as obesity.

## Methods

**Figure 1:**
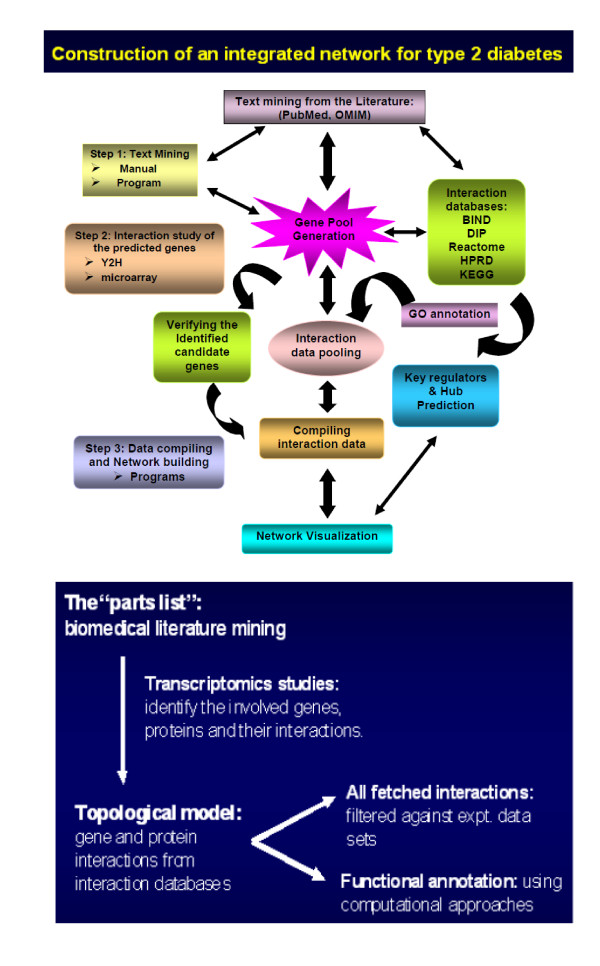
**Concept and overall strategy for generating the integrated network of T2D**.

The present work has developed gene interaction network models, comprised of high-throughput genomic and proteomic data for type 2 diabetes (T2D). The overall strategy of the integrated network model generation has been illustrated in Figure 1. The network model has been built in three successive stages. The first stage consisted of the "parts list" generation using biomedical literature mining. The second stage was the topological model generation by combining all the pooled genes and their interactions from all available interaction databases. The co-regulated and co-expression data fetched helped to identify the involved genes, proteins and their interactions. In third and final stage the fetched interaction data were filtered against experimentally validated interaction (e.g., yeast 2 hybrid, Chip- chip interaction, immuno-coprecipitation, arrays co-expression) data sets and the functional annotations were analyzed using computational approaches. By identifying the genes, the pathways they are involved in and the interactions they are part of, using a systems approach; the integrated networks of T2D have been generated considering all experimentally verified PPI data of the regulating genes. Finally from the integrated network systems the 'hubs' or key genes that play central roles in these diseases have been identified. Interestingly along the way of building up the network for T2D, gene networks of other related diseases like obesity and hypertension were also generated. The aim of this study was to identify the key switches of regulation and from that interpreting how these chronic diseases correlate and co-occur.

### Stage 1: Initial disease related gene pool generation through extensive text mining

The most important collection of scientific publications is PubMed http://www.ncbi.nlm.nih.gov/pubmed, which currently contains more than 19 million citations of biomedical articles from MEDLINE and Life Science journals. Citations include links to full-text articles from PubMed Central (PMC) http://www.ncbi.nlm.nih.gov/pmc/ or publisher web sites. Currently the number of publications is growing by more than 500K documents per year. PMC is the U.S. National Institutes of Health's (NIH) free digital archive of biomedical and Life Sciences journal literature serving the critical role of providing access to published literature toward the first step in the synthesis and translation of genomic research in an easy and comprehensive way.

PubMed basically supports keyword based searching and information Retrieval (IR). There have been a significant number of web-based text mining tools available to biologists to discover hidden relationships among biological entities. Some of them are quite effective in generating annotated relationships among graphs in the form of networks. AliBaba http://alibaba.informatik.hu-berlin.de is a tool that is capable of automatically and interactively extracting the most valuable information and graphically summarizing search results from PubMed [8]. It uses a dictionary-based approach for recognizing biomedical objects. Dictionaries consist of regular expressions depicting terms and spelling variations. The dictionaries are collected from different sources. To find associations among entities, AliBaba uses two different techniques in parallel: pattern matching and co-occurrence filtering. It mines relationships among cells, diseases, drugs, proteins, species and tissues based on a user query involving text key terms. Thus, AliBaba can be used to turn unstructured text into structured data records [8]. Specifically, for increasing the accuracy and efficiency for discovering relationships between important biological entities, e.g., protein-to-disease associations- the present study combines manually locating and querying information about disease genes entity from the biomedical literature together with automated computational tools to identify the genes and gene products by computationally capturing the related knowledge embedded in textual data.

### Stage 2: Using the pooled disease genes protein-protein interaction (PPI) maps construction

PPI maps have considerable impact on the discovery and synthesis of molecular networks. Thus, generating human protein interaction maps has become an important tool in biomedical research for the elucidation of molecular mechanisms and the identification of new modulators of disease processes.

**Figure 2:**
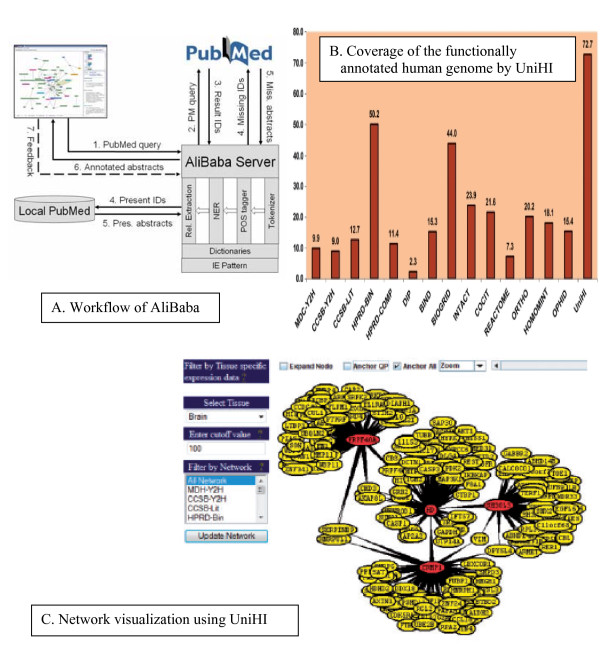
**Steps of network model generation**.

There have been a number of web-based tools available to biologists. The Unified Human Interactome database (UniHI, http://www.unihi.org) provides a comprehensive platform to query and access human protein-protein interaction (PPI) data [9]. The latest update of UniHI includes over 250,000 interactions between ~22,300 unique proteins collected from 14 major PPI sources [9]. However, this amount of data has its challenges. Even searches with a small number of query proteins can lead to large, highly connected and often unstructured networks (frequently referred to as 'hairballs'). UniHi has integrated separate PPI resources to provide a comprehensive platform for querying the human interactome (Figure 2). UniHI is not intended to replace single databases, but to offer a convenient single portal access to human protein interaction data for the biomedical research community. Additionally, it allows the identification of network topologies which would not be detectable if PPI resources were examined separately.

Pathway information can provide highly useful clues about the functions and dynamics of interactions. Especially for the elucidation of local network structures, knowledge of interrelated pathways can be of crucial importance. UniHI provides the possibility to examine the intersection of canonical pathways from Kyoto Encyclopaedia of Genes and Genomes (KEGG) with the extracted networks [10]. UniHI Scanner does not only show the proteins included in the pathway but also the KEGG annotation of the interactions (e.g. phosphorylation, activation or inhibition) between nodes [11]. In this way, it enables researchers to detect possible modifiers of pathways as well as proteins involved in the cross-talk between pathways and users can switch between the graphical display of the complete network and the intersection with selected pathways [11].

### Stage 3: Filtering and validation of the PPI data

Advances in recent genome-wide interactome projects have generated a wealth of PPI data. In order to understand the complexity, it is essential to extract meaningful information in the context of physiological systems. This necessitates identification of not only the function of individual proteins, but also to validate the physical interactions and biological processes in which they participate. The emergence of large scale protein-protein interaction maps has opened up new possibilities in systematically surveying and studying the underlying biological system. UniHI (the Unified Human Interactome database, http://www.unihi.org) integrates protein interaction data with pathway data from the Human Gene Expression Atlas [11]. In UniHI, PPI maps have been assembled using eight publicly available large-scale interaction maps: three literature-based, three orthology-based and two Y2H-based. Interactions in different maps are compared. For normalization of the intersections however, only the number of interactions between common proteins is used. Thus, the *interaction overlap *is defined as the average percentage of shared interactions between common proteins [12]. In UniHI, maps are subsequently clustered based on the interaction overlap. For all comparisons, it is notably larger than zero, which is the expected value for comparison of random maps. The observed concurrence of interaction maps does not occur merely by chance as it is validated by two permutation tests for pair-wise comparisons of graphs, where the observed overlap of interactions generated is highly significant for all comparisons (*P *< 0.01) [12]. To measure the conservation of connectivity between pairs of networks, UniHI correlates the number of interactions of proteins in the two networks using Spearman correlation for the set of common proteins [12]. A high correlation between two maps signifies that the interaction-rich (interaction-poor) proteins in one map are also interaction-rich (interaction-poor) in the other map. Finally, for the functional coherency of maps, UniHI employs the gene annotations available in GO [12]. Using UniHI, users can filter interacting proteins by requiring a minimum expression threshold, and the PPI network retrieved from UniHI can be reduced to include only highly expressed proteins or extended to include lowly expressed proteins. Additionally, the PPI resource to be queried can be specified. In this work, the fetched interactions data have been filtered against experimentally validated interaction (yeast 2 hybrid, Chip-chip interaction, immuno-precipitation, arrays co-expression) datasets.

### Integrated network visualization and identifying the 'hubs'

Flexible visualization is a crucial prerequisite for the display and evaluation of network structures. UniHI [9] provides users to switch between the graphical display of the complete network and the intersection with selected pathways. ***Cytoscape ***http://www.cytoscape.org/ is another open source bioinformatics software platform for visualizing molecular interaction networks and biological pathways and integrating these networks with annotations, gene expression profiles and other state data [13]. Although Cytoscape was originally designed for biological research, now it is a general platform for complex network analysis and visualization. Cytoscape *core *distribution provides a basic set of features for data integration and visualization. A variety of layout algorithms are available, including cyclic, tree, force-directed, edge-weight, and yFiles organic layouts. Additional features are available as *plugin**s ***which are available for network and molecular profiling analyses, new layouts, additional file format support, scripting, and connection with databases [13]. Cytoscape supports a lot of standard network and annotation file formats including: SIF, GML, XGMML, BioPAX, PSI-MI, SBML, OBO, and Gene Association. In this work along with UniHI [11], the interactions among the differentially expressed genes were visualized by using Cytoscape 2.5.1 software [13]. Candidate genes, which were common within various diseases, were identified. Network-neighbours around the fetched genes of T2D, HT, OBS and ROS been studied extensively to identify the 'hubs'. Genes with thirty or more gene degree in the PPI network were considered as hubs.

## Results

### Gene pool generation through extensive text mining

**Figure 3:**
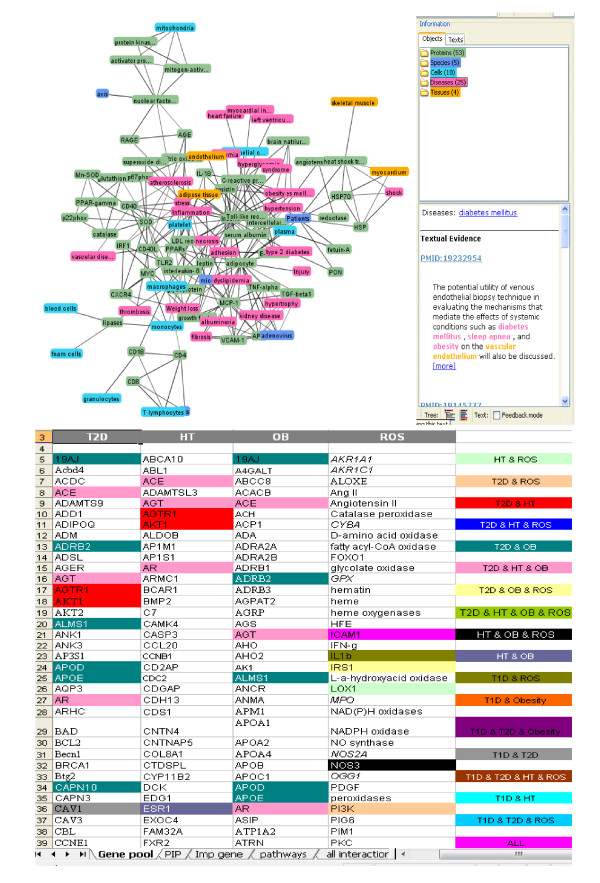
**Text mined data and the initial gene pool list**.

Candidate genes, responsible for the diseases (T2D, HT, OBS, ROS) were extracted and sorted out both manually (through literature review) and by using text-mining soft wares (Figure 3). At the beginning, the present work only focused on to understand the correlation between type 2 diabetes and hypertension (HT). But later on, ROS (Reactive Oxygen Species) and obesity (OBS) were also considered as they were found to be highly related to first two diseases. So, at the end candidate genes, responsible for all the diseases were pooled through text-mining. A number of genes for T2D, HT, OBS and ROS were identified. Among them the candidate genes were selected based on the following criteria's: i) Genes experimentally proved to be associated with any one of the above mentioned diseases, ii) Genes experimentally proved to be associated with two or more concerned diseases, iii) Genes predicted computationally to be associated with any individual disease, iv) Genes predicted computationally to be associated with two or more concerned diseases and v) Genes found in KEGG pathways database to be associated with any individual disease. So basically, a gene-disease network is generated by defining two genes as 'connected' if they have been studied for association with the same disease(s). The identified genes were further verified through literature review to get insight about their functions and also with KEGG for annotation in different biological pathways. These genes were also confirmed with Phenopedia (HuGEpedia: an integrated, searchable knowledge base of genetic associations and human genome epidemiology) [14] which provides the total number of reported genes that have genetic association with T2D, HT and other related diseases (Additional file 1, 2). After this first stage of text mining using literature databases, the number of genes identified and pooled for: Type 2 Diabetes (T2D) ~ 257, Hypertension ~100, Obesity ~239 and ROS ~55.

### Finding Protein-Protein Interaction partners (PIPs)

**Figure 4:**
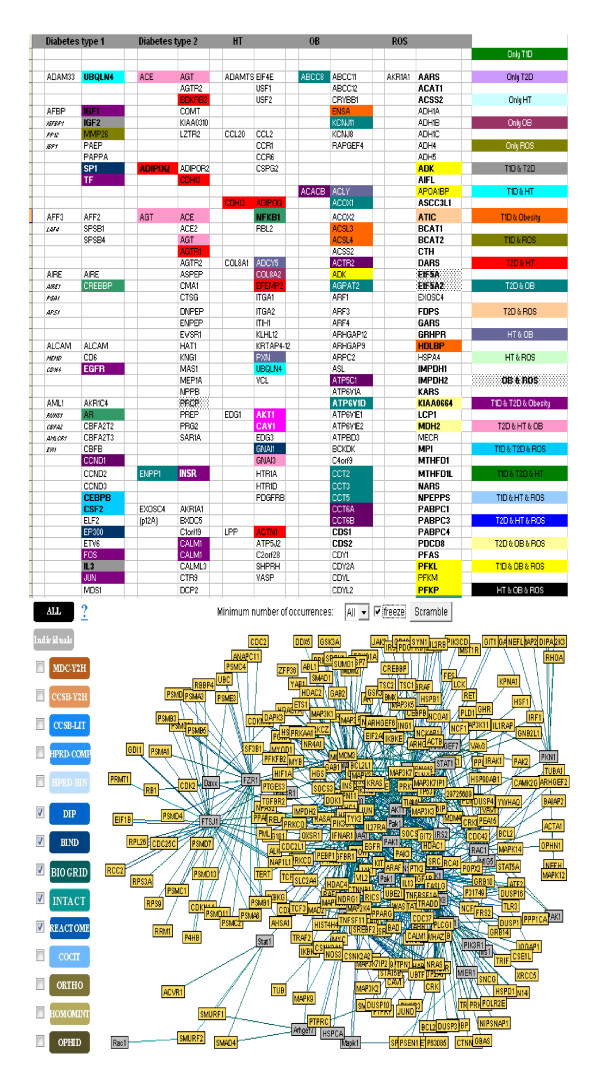
**Mined PPI data and graphical visualization using UniHI**.

Genes do not act as individual units; they collaborate in overlapping pathways, the co-regulation of which is a hallmark for the disease pathogenesis. A simple enrichment analysis has been applied in order to characterize the T2D set on the network level including pathways and protein-protein interactions. The computational approach used in this study was based on functional links derived from co-expression and co-regulation profiles. The co-expressed and co-regulated genes generally have a higher likelihood of having a direct physical interaction. The gene interaction data used to build the network was based on direct physical interactions that are either experimentally derived (e.g., Y2H or co-affinity purification) or computationally predicted. In order to integrate pathway information and to derive cellular network information on the selected genes, functional annotation from pathway databases such as KEGG, Reactome, BioCyc [15-17], protein-protein interaction databases such as BIND, DIP, REACTOME, COCIT, HPRD BIN, CCSB, MDC and IntAct was added [18]. To facilitate the findings, UniHi (*United Human Interactome*) [9], a web based PIP finding tool was used. The present work tried to find significantly interconnected sub-networks to interrogate whether the specific pattern formed by a pre-specified list of genes is significant (Figure 4). At this stage the number of significant candidate genes of inflammation related diseases were T2D ~1231, HT ~1000, OBS ~1845 and ROS ~417.

### Compiling of data and network visualization

Network subgraphs can be network modules, motif clusters, or other network neighborhoods. A network module can be defined as a subgraph consisting of highly interconnected nodes that may fulfill a particular biological function [19-21]. At this stage of network building, the pooled important common genes for various diseases were integrated into network models and visualized using UniHI [18], Cytoscape [13]. The models have been generated through several stages of screening where in subsequent steps the pooled data have been filtered, verified for authentication and most importantly, validated with experimentally derived data to make the output graph generated for different diseases meaningful and interpretable. This work constructed four disease networks (Additional file 2), each containing a set of proteins that are associated with the diseases. The hub nodes form the backbone of a network, they are considered to be an important measurement for the similarity between protein interaction networks. Moreover, backbone network has also been shown to be highly conserved in maintaining biological function of the cell [22]. Within the networks the "hubs" are defined as the protein nodes with degrees ≥ 15 in a disease network [22]. The generated model of T2D has predicted the highest number of hub proteins among the four diseases we studied so far, probably due to the fact that it has the highest number of genes that were used to construct the disease network (Additional file 3).

### Molecular linkage among T2D, HT, OBS and Inflammation

**Figure 5:**
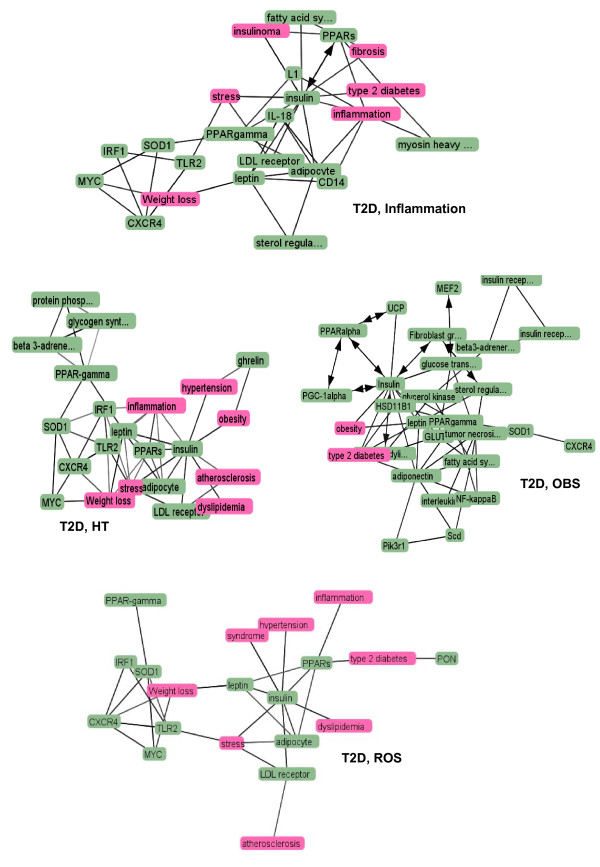
**Cross-linkage between various diseases**.

**Figure 6:**
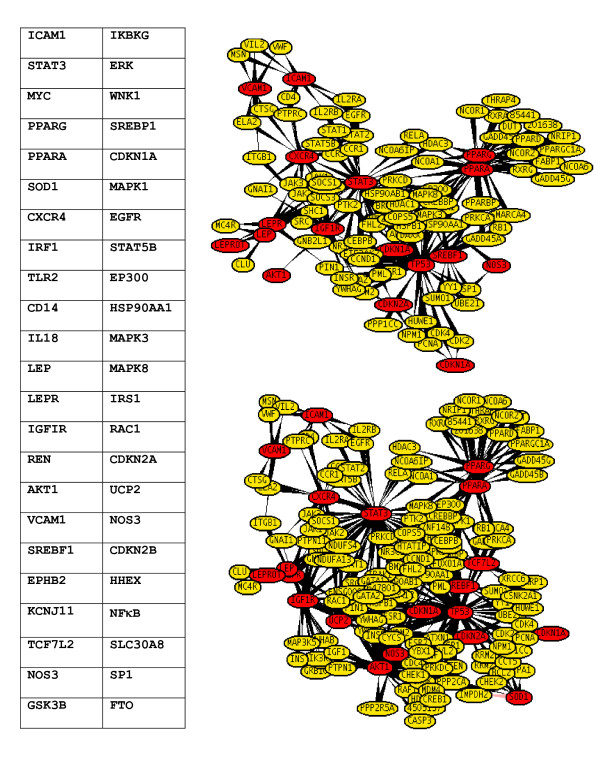
**Main 'hub' genes and connections**.

**Figure 7:**
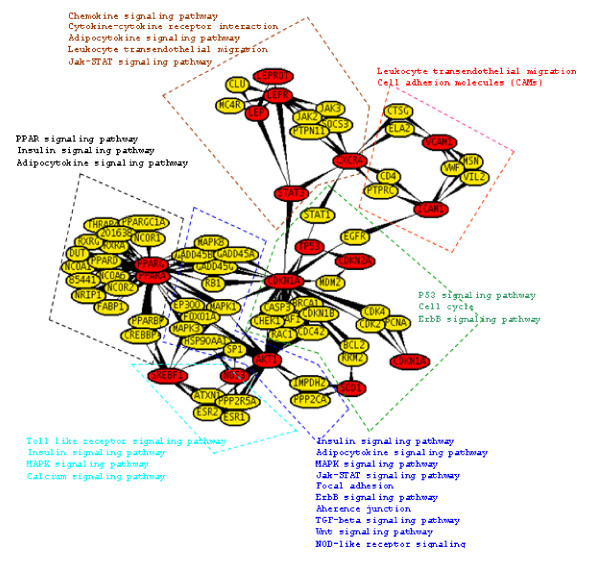
**Important signaling pathways integration with gene network**.

**Figure 8:**
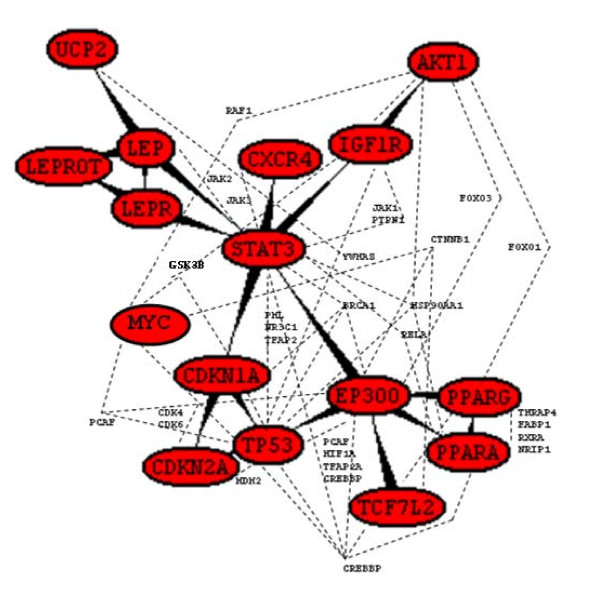
**Gene regulatory network around the highly inter-connected key hubs**.

To investigate the molecular cross-talk within these interrelated disease mechanisms, the individual disease networks were looked thoroughly at their 'hubs' to understand the underlying connections between genes and the disease mechanism. This was done in three phases. First, we developed the gene-disease network using the text miner Ali-Baba [8] (Figure 5). Thorough analyses of the gene patterns and their relatedness within different diseases, the way they were connected, the cross talk - a gene list was generated containing the connections in all four diseases. In the second phase, the PPI patterns around the selected hub genes were studied extensively using UniHI [9], validated the connections using Gene Ontology (GO) [23] and Genopedia and Phenopedia [14] databases for annotation (Figure 6 and Additional file 4). And finally in the third phase, the selected genes were cross-validated and clustered using the pathway database KEGG [10] (Figure 7). During the whole process a list of 42 co-regulated genes were analysed and these candidate genes were significantly clustered in 19 pathways. The candidate genes were looked for common interaction partners and the highly integrated gene regulatory network was generated (Figure 8).

## Discussion

In this study, a text mining approach was used to identify the disease-related genes of T2D and HT. Along the way of querying, genes associated with oxidative stress (reactive oxygen species, ROS) and OBS was also included based on- the two diseases are 'connected' if they have been studied for association with the same gene(s) hypothesis. These data were further analysed using various novel bioinformatics tools and resources to identify the key candidate hub genes associated with various diseases.

Literature-based annotation analyses showed that all these diseases T2D, HT, OBS are somehow correlated with oxidative stress and inflammation (Figure 5). Further PPI studies, generated the disease-gene network within T2D, HT, OBS, ROS and Inflammation. In this work, the genes for inflammation were never pooled separately but from the cross-talk between the networks of T2D, HT and OBS, the strong co-regulated inflammation genes were identified. Although at this point it was not completely understandable how ROS or inflammation plays role in disease mechanism. Further pathway-based investigation, using both Genopedia [14] and KEGG [15] provided more insights about the roles of these genes in association with different disease phenotypes. This work predicted the involvement of about 20 enriched pathways, some of which are very commonly involved in diabetes and/or obesity like the insulin signaling, PPAR signaling, Adipocytokine signaling, MAPK signaling, Jak-STAT signaling pathways. But along them, the involvement of Wnt signaling, p53 signaling, ErbB signalling, TGF-beta signalling, Focal adhesion, toll-like receptor signaling pathways were predicted, whose associations are more widely linked with cancer metastasis (Figure 8 and Additional file 4).

From pathway analysis (Figure 7), the involvement of p53 or Wnt signaling pathway shed new light to focus at the PPI networks. After further filtering of the data (through network and/or PubMed interaction partners, IPs) along with cross validation through literature annotation, the most interesting 46 candidate 'hub' genes were identified. Careful investigation around these 'hubs' provide more meaningful insights about the cross- talk within gene-disease networks in terms of disease phenotype association with ROS and inflammation (Figure 6). From network analysis, common IPs revealed that all the diseases T2D, HT, OBS are linked via two common gene regulatory cascades: (i) EP300/TP53/MYC/CDKN1A/STAT3/CXCR4 and (ii) PPARG/PPARA/IGF1R/AKT1/LEP/LEPR. Basically, these regulatory routes connect insulin signaling pathway through oxidative stress and inflammation. From investigating the 'hub' gene network connections and broadly studying the interconnection, one hypothesis could be, the components of the second routes can only be regulated through the first or vise-versa (Figure 8).

To further investigate the hypothesis, and to address how the connections regulated within the network that could be associated with genesis of inter-connections between various diseases, the 'gene-links' associating two or more hubs were identified. In course of the analysis, regulations around EP300, PPARG, PPARA, STAT3, IGF1R, CXCR4, MYC, TP53, CDNK1A, CDKN2A, PCAF, HIF1A, CREBBP, CEBPB, RELA, HSP90AA1, MAPK3, MAPK8, JAK2, JAK3, PTPN11, TFAP2A, LEP, TCF7L2, CTNNB1, FOXO1A, FOXO3A and GSK3B seem to play crucial roles in the regulation of T2D and other related disorders (Figure 8). Interestingly, the genes like GSK3B, PCAF, HIF1A, CREBBP, CEBPB, RELA, TCF7L2, CTNNB1, MYC, TP53, CDNK1A are known as important role players in Wnt and p53 signaling pathways, which would provide a putative link between T2D and certain types of cancer.

The molecular events leading to β-cell failure in the diabetic environment, in particular high levels of glucose and free fatty acids, exert toxic effects on the β-cell [24]. A number of signaling pathways have been implicated in β-cell failure, including insulin signaling [25] and oxidative stress [26]. Ectopic overexpression of the Wnt target gene c-myc in mice has been shown to cause β-cell apoptosis and diabetes [27]. Also extensive recent investigations have revealed the existence of molecular crosstalk between insulin and Wnt signaling pathway [28-32]. A number of genetic studies have confirmed that TCF7L2, a direct downstream target of β-catenin of the Wnt pathway, to be involved in β-cell dysfunction and the etiology of T2D [24,33]. There is clear evidence that TCF7L2 regulates insulin secretion rather than insulin action.

A central feature of the Wnt/β-catenin pathway is the regulation of cytosolic β-catenin protein levels via a destruction complex containing glycogen synthase kinase-3b (GSK-3b). GSK-3B has long been known as an important mediator for impaired insulin action on peripheral tissue and in the development of insulin resistance [34]. However, only recently, studies have shed light on the growth regulatory properties of GSK-3B in β-cells. Two elegant studies from the same group reported recently that GSK-3B overexpression in mice induces β-cell mass restriction and the development of diabetes [35] and that the genetic disruption of GSK-3B in β-cells results in increased β-cell mass in those transgenic mice [36].

Importantly, β-catenin is able to interact with the FoxO family of transcription factors, which defend against oxidative stress by stimulating the transcription of oxidant scavenging enzymes such as superoxide dismutase and catalase [37]. Since FOXO and TCF proteins compete for a limited pool of β-cat, enhanced FOXO activity during ageing and oxidative stress may attenuate Wnt-mediated activities. The decrease of β-catenin not only attenuates Wnt signaling but also unleashes the expression of PPARγ, which is normally suppressed by β-catenin [38-40]. Oxidative stress activates the FoxO family of transcription factors, which in turn attenuate β-catenin/TCF-mediated transcription, leading to derepression of PPARγ transcription. The increase in PPARγ levels serves as an additional β-catenin sink by sequestering it and activating its proteasomal degradation [41].

Adipogenesis is also affected by oxygenation, low oxygen tensions activate HIF (hypoxia inducible factor)-1α, inhibiting differentiation by repressing PPARγ (peroxisome proliferator-activated receptor γ) [42] whereas higher oxygenation tends to induce differentiation [43-45]. HIF-1α has been shown to compete with TCF4/TCF7L for direct binding to β-catenin [46]. A recent work showed that Wnt signaling (which is up-regulated in high oxygen) has been associated with increased β-cell proliferation [47]. Thus, the increase in Wnt activity associated with higher oxygen availability might have the twofold effect of increasing (a) endocrine cell mass by self replication and (b) exocrine cell mass by progenitor cell expansion [48].

Another study showed the involvement of p53 expression in adipose tissue is crucial in the development of insulin resistance. The work showed in animal model with T2D-like disease, excessive calorie intake led to the accumulation of oxidative stress in the adipose tissue and promoted increased activity of senescence-associated β-galactosidase, increased expression of p53 and increased production of pro-inflammatory cytokines. Conversely, up-regulation of p53 in adipose tissue caused an inflammatory response that led to insulin resistance [49]. It has also been reported that production of reactive oxygen species (ROS) is selectively increased in the adipose tissue of obese mice and that increased oxidative stress in fat is a key mechanism underlying the occurrence of insulin resistance related to obesity [50]. The ROS-induced p53 activation causes NF-κB-dependent induction of inflammatory cytokines and thus accelerates the development of diabetes.

Once oxidative stress is initiated it affects multiple systems. By reaction of ROS with NO, oxidative stress is increased while NO is diminished, thus promoting inflammation and endothelial dysfunction [51]. Specifically, ROS were implicated in mitogen-activated protein kinase (MAPK) pathways, which induce activation of various nuclear transcription factors, such as nuclear factor (NF)-κB, activator protein (AP)-1, hypoxia-inducible factor (HIF)-1a, sterol regulatory element binding proteins (SREBPs) and GATA-4 [52-54]. NF-κB participates in obesity and the metabolic syndrome; it induces inflammatory and atherosclerotic consequences. Obesity increases the formation of reactive oxygen species in fat cells, and ultimately results in activation of the p53 tumor suppressor, inflammation and the promotion of insulin resistance [55]. Also, insulin resistance is affected by oxidative stress and, when combined with up regulated NF-κB activity, may promote type 2 diabetes. Conversely, hyperglycaemia was also shown to trigger increased formation of ROS via glucose auto-oxidation. Accordingly, consumption of a high free-glucose diet promoted the development of oxidative stress [56-58]. HIF-1a, is also associated co morbidities such as hypertension [59,60] and hyperlipidaemia [61]. High glucose concentration up-regulates SREBP-1c and insulin resistance [62]. However, none of these mechanisms are yet fully elucidated [63].

### Proposed hypothetical model of mechanism

**Figure 9:**
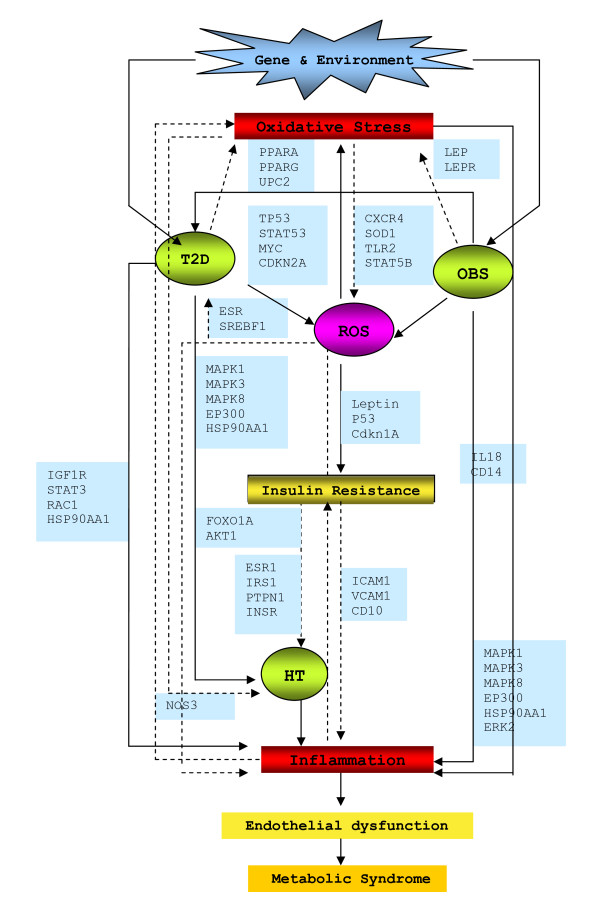
**Proposed hypothetical model of T2D mechanism**.

Based on an elaborate study of the key 'hubs' regulations, their association, along with detailed literature reviewing - in this work we put the puzzle pieces together and proposed a hypothetical mechanism for co-regulation of various inflammatory diseases like T2D, HT and OBS (Figure 9). From the generated regulatory networks around key 'hub' genes it is very much visible that via EP300 the main pathways like insulin signaling, PPAR signaling, calcium signaling, adipocytokine signaling, Jak-STAT signaling pathway, MAPK signaling pathways that are well recognised in association with disease like T2D, OBS and HT. Interestingly, the analyses of the regulatory cascades showed that the genes involved in main disease pathways are all connected and regulated via the genes involved in Wnt-signaling and p53 signaling pathways.

In detailed study of the gene-disease network and the regulation around the 'hubs', this work suggests EP300/TP53/CDKN1A/STAT3/TCF7L2/MYC/CXCR4 is the main regulatory cascade that generates the highly connected, co-regulated network of insulin signaling which in turn, could be responsible for the genesis of molecular cross-talk within various diseases. It has been suggested that insulin signaling crosstalk with Wnt signaling, due to the existence of common downstream target genes and the shared negative mediator GSK-3 [33]. However, mechanisms underlying this potential crosstalk still remain unclear [33]. β-catenin, an intracellular signaling molecule is essential for Wnt activation. Without Wnt signal, β-catenin, is constantly phosphorylated and thereby inactivated by GSK3B, is eventually degraded to prevent its accumulation. Inactivation of GSK3B, in turn can no longer phosphorylate β-catenin. This leads to nuclear translocation of β-catenin and subsequently, which coactivates TCF/LEF transcription factors [64]. Our regulatory gene- network could fit all those observations and explain a bit more these underlying molecular mechanisms.

Wnts induce glucose-stimulated insulin secretion and β-cell proliferation [65]. This signaling system might senses insulin sensitivity via the regulator gene GDK3B inactivation/activation (phosphorylation/dephosphorylation) by increasing insulin effects through AKT1/IGF1R/STAT3/MYC/CDKN1A/TP53/EP300 cascade via activating Akt/PKB and inhibiting the MAPK pathways (Figures 7, 8 and 9). In the absence of insulin, FOXOs upregulate the expression of a set of target genes via AKT1/IGF1R/CXCR4/STAT3/EP300/PPARG cascade, thereby promoting cell cycle arrest, stress resistance and apoptosis [65]. In the presence of insulin, FOXOs are phosphorylated by AKT/PKB protein kinase and stay in the cell cytosol [66]. In contrast to the effect on insulin signaling, oxidative stress (ROS) induces the activation of FOXO signaling [67]. This might be due to the activation via the Jak/STAT signaling pathway [68]. T2D, age, obesity- all increases ROS that in turn increases lipid oxidation, via ROS/FOXO/PPARG/PPARA/β-catenin regulation leads to PPARG activation and repression of Wnt-signaling. So, when PPARG activates it attenuates Wnt-signaling, which induces lipid oxidation that in turn increases ROS. Increased oxidative stress diminished Wnt signaling that may leads to β-cell destruction via FOXO apoptosis [69]. Regulatory gene network (Figure 8) suggests that one possibility could be via CREBBP regulation on MYC/TP53/EP300/TCF7L2 cascade, the system might sense signal to FOXO. Since FOXO and TCF compete for limited pool of β-cat, enhanced FOXO activity during oxidative stress may attenuate WNT- mediated activation [70]. Thus, oxidative stress lead to FOXO mediated gene transcription and reduced TCF mediated gene transcription.

Conversely, activation of Wnt pathway through inactivation of GSK3B, stabilise β-catenin, that regulates β-cell proliferation via TCF7L2, a down stream effector of this cascade [69]. This in turn coactivates PPARG mediated transcription on the glucokinase gene promoter. Glucokinase is the key regulator of glucose-sensing in pancreatic beta-cells, thereby offering a model for the adipocyte-induced hyper secretion of insulin [71]. Although TCF7L2 expression was positively correlated with the expression of INS, which encodes insulin, it was inversely correlated with glucose-stimulated insulin release [72]. There is clear evidence that TCF7L2 regulates insulin secretion rather than insulin action [65]. Thus, β-catenin interaction with TCF7L2 with nuclear co-activators, such as EP300, results in the stimulation of Wnt or β-cat/TCF downstream target gene transcription [73-75]. From the gene regulation network, it can be seen that insulin may also stimulates the transcription via MYC, a known downstream target of Wnt signaling, via a PI3K-dependent but Akt/GSK3B-independent mechanism, via IGF1R/MYC/TP53/EP300 regulatory cascade. Indeed, several other studies have also reported the stimulatory effect of insulin or IGF1 on β-cat nuclear localization and cat/TCF mediated gene transcription [29,33,76,77]. Thus, fitting all these evidences in the proposed hypothetical model (Figure 9), it can be suggested that insulin signaling may crosstalk with Wnt signaling, due to the existence of common downstream target genes, the shared negative mediator GSK3B, and insulin resistance are capable of explaining the association of T2D co-occurrences with other inflammatory disease like HT, and OBS.

T2D and/or OBS can develop independently, due to genetic, behavioral or lifestyle-related variables but both T2D and OBS lead to oxidative stress generation [78]. The pathogenic mechanisms by which diabetes and oxidative stress induce inflammation are not certain at the present time [79]. But the predicted model, at least provided much more logical explanation to fit what so far been known about the inflammation mediated consequences. Excessive calorie intake led to the accumulation of oxidative stress with T2D that can promote increased activity of senescence-associated β-galactosidase, increased expression of p53 and increased production of proinflammatory cytokines. Conversely, upregulation of p53 in adipose tissue can cause an inflammatory response that led to insulin resistance [79]. Also oxidative stress through TCF7L2/NF-kB/MAPK/HIF1A/SREBP regulatory cascade via insulin, MAPK and Calcium signaling pathways, may activate inflammatory responses than in turn causes insulin resistance. Hyperglycemia could lead to increase lipid oxidation and endothelial dysfunction regulated through PPARG/STAT3/CXCR4/ICAM/VCAM cascade via Chemokine, Adipocytokine and Jak-STAT signaling pathways that could cause increase expression ICAM1, VCAM1 levels which in turn increases inflammation and obviously, increases oxidative stress [80,81]. Once oxidative stress is initiated it affects multiple systems-via AKT1/NOS3 regulation. Increased oxidative stress can diminish NO production, thus promoting inflammation and endothelial dysfunction [51]. Inflammatory pathway activation will lead increased expression of adhesion molecules, cytokines and associated with increased risk of HT. Obesity, insulin resistance and hypertension commonly cluster with other risk factors for CDV or chronic kidney disease to form the metabolic syndrome [65].

## Conclusion

It can be hypothesised from the generated model (Figure 9) that oxidative stress is a direct cause for the Wnt pathway activation via the high glucose-induced β-catenin activation through a highly inter-connected gene regulatory cascade, where EP300 plays a role as a key regulator in T2D. Even though these predictions need further experimental validation, the hypothetical model presented in this study could be a starting point to visualize in a systems approach understanding the molecular cross- talk between diseases. The findings provide a novel integrated approach for understanding the pathogenesis of various co-associated diseases by combining the power of pathway analysis with gene regulatory network evaluation. The integrated network model developed by this work provides useful functional linkages among groups of genes and thus addressing how different inflammatory diseases such as obesity, diabetes, and hypertension are connected and propagated at genetic level. It is anticipated that derived models will be of great benefit to a wide research audience, including those involved in disease biomarker identification and drug development.

## Competing interests

The authors declare that they have no competing interests.

## Authors' contributions

Jesmin, MSMR and HJ coordinated the text mining and information extraction, Jesmin, MSMR mined and computed the interaction data, Jesmin, HJ, JB-R, and RH analysed and interpreted the result, drafted and revised the manuscript. All authors have read and approved the final manuscript.

## Pre-publication history

The pre-publication history for this paper can be accessed here:

http://www.biomedcentral.com/1755-8794/3/45/prepub

## Supplementary Material

Additional file 1 **Statistics of the reported genes associated with T2D, HT, OBS and ROS**. Click here for file

Additional file 2 **Complete 'text-mined' data set**. Click here for file

Additional file 3 **Predicted integrated network model of the four diseases (using Cytoscape)**. Click here for file

Additional file 4 **Gene annotation using GO, Genopedia and Phenopedia**. Click here for file
