# Supplementary material for: Gene regulatory network reveals oxidative stress as the underlying molecular mechanism of type 2 diabetes and hypertension
Source: BMC Med Genomics. 2010 Oct 13;3:45. doi: 10.1186/1755-8794-3-45 (PMC2965702; doi:10.1186/1755-8794-3-45)
Supplement: Additional file 1 — Statistics of the reported genes associated with T2D, HT, OBS and ROS. [file 1755-8794-3-45-S1.DOC]

| **Disease** | **Total no of genes** | **Total no of Publications** | **Meta-Analyses**  **data** | **Genome wide association study (GWAS)** |
| --- | --- | --- | --- | --- |
| **T2D** | 675 | 1969 | 107 | 46 |
| **HT** | 1001 | 2680 | 52 | 25 |
| **OBS** | 644 | 2028 | 69 | 29 |
| **ROS** | 734 | 1112 | 11 | 3 |
