# Supplementary figures and images for: Gene regulatory network reveals oxidative stress as the underlying molecular mechanism of type 2 diabetes and hypertension
Source: BMC Med Genomics. 2010 Oct 13;3:45. doi: 10.1186/1755-8794-3-45 (PMC2965702; doi:10.1186/1755-8794-3-45)

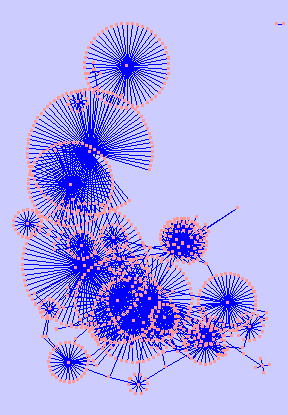


**T2D**


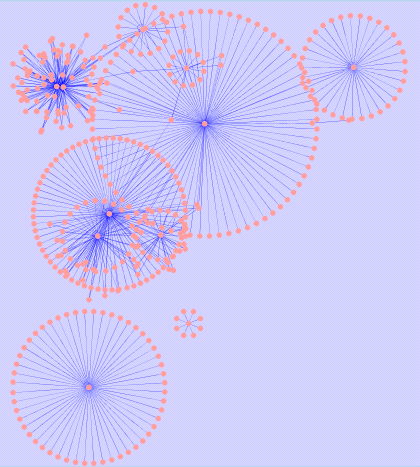


**OBS**


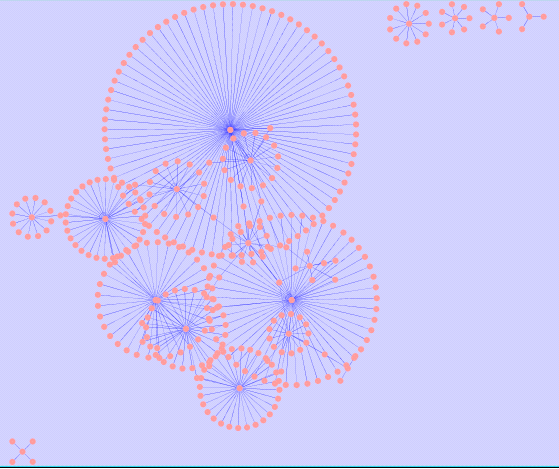


**HT**


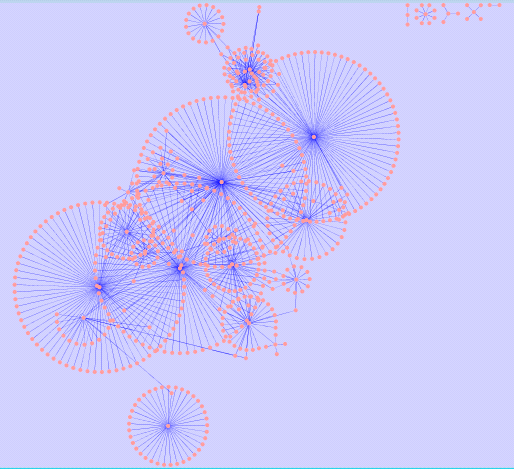


**ROS**

Supplement: Additional file 3 — Predicted integrated network model of the four diseases (using Cytoscape). [file 1755-8794-3-45-S3.DOC]
