# Supplementary material for: Gene regulatory network reveals oxidative stress as the underlying molecular mechanism of type 2 diabetes and hypertension
Source: BMC Med Genomics. 2010 Oct 13;3:45. doi: 10.1186/1755-8794-3-45 (PMC2965702; doi:10.1186/1755-8794-3-45)
Supplement: Additional file 4 — Gene annotation using GO, Genopedia and Phenopedia. [file 1755-8794-3-45-S4.DOC]

| GENE ID | Description/ Location | No. of Disease terms | Total Publications | Important Disease Terms | Genes involved in KEGG Pathways |
| --- | --- | --- | --- | --- | --- |
| ICAM1  **GeneID:** 3383 | intercellular adhesion molecule 1  Chromosom19; **(**19p13.3-p13.2) | 123 | 145 | Crohn Disease, Diabetes Mellitus, Type 2, Inflammation, Cardiovascular Diseases, Colitis, Ulcerative, Myocardial Infarction, Coronary Disease, Alzheimer Disease ,Arthritis, Rheumatoid, Inflammatory Bowel Diseases, Diabetic Retinopathy, Brain Ischemia, Stroke, Diabetes Mellitus, Type 1, Respiratory Syncytial Virus Infections, Multiple Sclerosis, Recurrence, Obesity, Celiac Disease, Atherosclerosis, Behcet Syndrome, Disease Susceptibility, Endometriosis, Graves Disease, Hypertension, Diabetic Nephropathies, Insulin Resistance, Kidney Diseases, Kidney Failure, Chronic, Biliary Atresia, Bronchiolitis, Viral, Asthma, Acute Disease, Cholangitis, Sclerosing, Chronic Disease, Coronary Arteriosclerosis Colorectal Neoplasms , Osteoporosis, Lung Neoplasms, Malaria, Falciparum, Melanoma, Schizophrenia, Skin Neoplasms, Vasculitis, Venous Thrombosis, Vertebral Artery Dissection, Vision Disorders, Waldenstrom Macroglobulinemia, Shock, Septic, Sarcoidosis syndrome, Systemic Inflammatory Response Syndrome, Temporal Arthritis, Thrombosis, Trachoma, Urinary Tract Infections, Respiratory Distress Syndrome, Adult, Multiple System Atrophy, Myocardial Reperfusion Injury, Neoplasms, Neoplasms, Glandular and Epithelial , Metabolic Syndrome X, Migraine Disorders, Lymphoma, Large B-Cell, Diffuse, Lymphoma, Non-Hodgkin, Lymphoproliferative Disorders, Malaria, Ovarian Neoplasms, Pancreatitis, Periodontitis, Peripheral Vascular Diseases, Pneumonia, Polymyalgia Rheumatica, Postoperative Complications, Premature Birth, Prostatic Neoplasms, Pseudoxanthoma Elasticum, Pulmonary Fibrosis, Purpura, Schoenlein-Henoch, Q Fever, Common Cold , Community-Acquired Infections, Constriction, Pathologic, Coronary Artery Disease, Coronary Restenosis, Cicatrix, Breast Neoplasms, Bronchiolitis, Cerebrovascular Accident, Cerebrovascular Disorders, Chlamydia Infections, Albuminuria, Astrocytoma, Amyotrophic Lateral Sclerosis, Anemia, Sickle Cell, Angina Pectoris, Arterial Occlusive Diseases, Calcinosis, Carcinoma, Non-Small-Cell Lung, Carcinoma, Squamous Cell, Autoimmune Diseases, Leukemia, Leukemia, Promyelocytic, Acute, Ischemia, Dementia, Vascular, Diabetes Complications, Infant, Premature, Diseases, Infection, Encephalitis,Graves Ophthalmopathy, Hepatitis B, Chronic, Hodgkin Disease, Hypercholesterolemia, Hyperlipidemias, Hypersensitivity, Erythema Nodosum, Esophageal Neoplasms, Fatigue Syndrome, Chronic Fibrosis, Gastrointestinal Hemorrhage, Gastroschisis,Genetic Diseases, Inborn | Cell adhesion molecules (CAMs),  Leukocyte transendothelial migration,  Viral myocarditis  Natural killer cell mediated cytotoxicity |
| STAT3  **GeneID:** 6774 | signal transducer and activator of transcription 3  **Chromosome:** 17; **Location:** 17q21.31 | 30 | 29 | Crohn Disease, Asthma, Insulin Resistance, Bronchiolitis, Viral ,Obesity,Respiratory Syncytial Virus Infections, Colitis Ulcerative, Thyroid Neoplasms, Tooth Abnormalities, Rectal Fistula, Carcinoma, Renal Cell, Cardiovascular Diseases, Cleft Lip, Cleft Palate, Depressive Disorder, Diabetes Mellitus, Type 1,Disease Progression, Disease Susceptibility, Eczema, Fatty Liver , Job's Syndrome, Kidney Neoplasms, Lymphoma, Non-Hodgkin, Multiple Sclerosis, Relapsing-Remitting Neoplasm Metastasis, Autoimmune Diseases, Breast Neoplasms, Infant, Premature, Diseases, Inflammation | Jak-STAT signaling pathway,  Chemokine signaling pathway,  Adipocytokine signaling pathway,  Pathways in cancer, Pancreatic cancer,  Acute myeloid leukemia |
| MYC  **GeneID:** 4609 | v-myc myelocytomatosis viral oncogene homolog  **Chromosome:** 8; **Location:** 8q24.21 | 22 | 20 | Breast Neoplasms, Lung Neoplasms, Neoplasms, Prostatic Neoplasms, Thyroid Neoplasms, Waldenstrom Macroglobulinemia, Stomach Neoplasms, Thyroid Diseases, Osteoporosis, Hodgkin Disease, Kidney Failure, Chronic Lymphoma, B-Cell, Lymphoma, Follicular, Lymphoma, Large B-Cell, Diffuse Lymphoproliferative Disorders, Metabolic Syndrome X, Mouth Neoplasms, Carcinoma, Non-Small-Cell Lung, Carcinoma, Squamous Cell, Cardiovascular Diseases, Diabetes Complications, Alzheimer Disease |  |
| PPARG  **GeneID:** 5468 | peroxisome proliferator-activated receptor gamma  **Chromosome:** 3; **Location:** 3p25 | 137 | 445 | Diabetes Mellitus, Type 2, Obesity, Insulin Resistance, Hypertension, Metabolic Syndrome, Inflammation, Polycystic Ovary Syndrome, Diabetes Mellitus, Body Weight, Birth Weight, Colorectal Neoplasms, Cardiovascular Diseases, Myocardial Infarction, Weight Gain, Glucose Intolerance, Diabetes Mellitus, Type 1, Coronary Disease, Diabetic Nephropathies, Colonic Neoplasms, Weight Loss , Obesity, Morbid, Alzheimer Disease, Adenocarcinoma, Breast Neoplasms, Diabetic Retinopathy , Disease Progression, Diabetes Complications, Diabetes, Gestational, Lung Neoplasms, Rectal Neoplasms, Hyperinsulinism, Kidney Failure, Chronic, Coronary Arteriosclerosis, Arteriosclerosis, Brain Ischemia, Adenoma, Neoplasms, Prostatic Neoplasms, Schizophrenia, Osteoporosis, Stomach Neoplasms, Stroke, Peripheral Vascular Diseases, Overweight, Recurrence, Lipid Metabolism, Inborn Errors, Neuropsychological Tests, Hyperlipidemia, Familial Combined, Hyperlipidemias, Hypertriglyceridemia, Hyperglycemia, Albuminuria, Arthritis, Rheumatoid, Asthma, Carcinoma, Carcinoma, Non-Small-Cell Lung, Carotid Artery Diseases, Cerebrovascular Accident, Carcinoma, Squamous Cell, Cognition Disorders, Colitis, Ulcerative, Diabetic Angiopathies, Disease Susceptibility, Dyslipidemias, Glucose Metabolism Disorders, Helicobacter Infections, Edema, Endometrial Neoplasms, Endometriosis, Fatty Liver, Femur Head Necrosis, Gallstones, Gastritis, Esophageal Neoplasms, Hepatitis, Toxic, Hirsutism, HIV-Associated Lipodystrophy Syndrome, Hyperandrogenism, Graves Disease, Graves Ophthalmopathy, Dyspepsia, Diabetic Neuropathies, Drug Toxicity, Crohn Disease, Dementia, Colonic Polyps, Constriction, Pathologic, Chest Pain, Chronic Disease, Carcinoma, Renal Cell, Carcinoma, Basal Cell, Atherosclerosis, Biliary Tract Neoplasms, Breast Diseases, Arthritis, Psoriatic, Alcoholism, Adenomatous Polyps, Amyotrophic Lateral Sclerosis, Angina Pectoris, Variant, Arrhythmogenic Right Ventricular Dysplasia, Kidney Diseases, Lupus Erythematosus, Systemic, Lymphoma, Non-Hodgkin Melanoma, Mental Status Schedule, Metabolic Diseases, Hypertrophy, Hypertrophy, Left Ventricular Glomerulonephritis, IGA Neoplasms, Glandular and Epithelial, Orbital Diseases, Osteoarthritis, Knee, Kidney Neoplasms, Leiomyoma, Neoplasm Recurrence, Local Microsatellite Instability, Multiple Myeloma, Multiple Sclerosis, Renal Insufficiency, Chronic Sarcoidosis, Skin Neoplasms, Starvation, Psoriasis, Psychiatric Status Rating Scales, Pancreatic Neoplasms, Peptic Ulcer, Osteoporosis, Postmenopausal Ovarian Neoplasms, Pre-Eclampsia Prediabetic State, Premature Birth, Prenatal Exposure Delayed Effects, Thinness, Thyroid Neoplasms, Urinary Bladder Neoplasms, Uterine Neoplasms | PPAR signaling pathway, Insulin signaling pathway, Adipocytokine signaling pathway,  Thyroid cancer, Huntington's disease, Pathways in cancer |
| PPARA  **GeneID:** 5465 | peroxisome proliferator-activated receptor alpha  **Chromosome:** 22; **Location:** 22q13.31 | 46 | 90 | Diabetes Mellitus, Type 2, Obesity, Coronary Arteriosclerosis, Hypertension, Hypertriglyceridemia, Hyperlipidemias, Coronary Disease, Cardiovascular Diseases, Atherosclerosis, Myocardial Infarction, Body Weight, Alzheimer Disease, Diabetes Mellitus, Fatty Liver, Disease Progression Dyslipidemias, Edema, Hyperlipidemia, Familial Combined Hypertrophy, Left Ventricular, Insulin Resistance, Arteriosclerosis, Diabetes Complications. Obesity, Morbid, Osteoporosis, Peripheral Vascular Diseases, Prostatic Neoplasms, Psoriasis, Psychomotor Performance, Syndrome, Thrombosis, Neoplasms, Diabetic Angiopathies, Dementia, Coronary Artery Disease, Adenocarcinoma, Arterial Occlusive Diseases, Brain Ischemia Breast Neoplasms | Adipocytokine signaling pathway,  PPAR signaling pathway, |
| SOD1  **GeneID:** 6647 | superoxide dismutase 1  **Chromosome:** 21; **Location:** 21q22.11 | 36 | 54 | Myotrophic Lateral Sclerosis, Breast Neoplasms, Disease Progression, Prostatic Neoplasms, Diabetes Mellitus, Type 2, Diabetic Angiopathies, Pulmonary Disease, Chronic Obstructive Lymphoma, Non-Hodgkin, Motor Neuron Disease, Multiple Sclerosis, Neuroblastoma, Neuropsychological Tests, Occupational Diseases, Pre-Eclampsia, Schizophrenia, Schizophrenic Psychology, Sensory Thresholds, Urologic Diseases, Weight Gain, Encephalitis, Japanese, Diabetic Nephropathies, Diabetic Retinopathy, Glioblastoma, Glioma, Hemorrhage, Hepatitis, Toxic Lung Neoplasms, Carcinoma, Ductal, Breast, Carcinoma, Lobular Cognition Cognition Disorders, Dementia, Diabetes Mellitus, Type 1, Brain Neoplasms, Albuminuria, Alzheimer Disease | Neurodegenerative diseases,  Amyotrophic lateral sclerosis (ALS), Huntington's disease,  Prion diseases |
| CXCR4  **GeneID:** 7852 | Chemokine (C-X-C motif) receptor 4  **Chromosome:** 2; **Location:** 2q21 | 19 | 24 | HIV Infections, Lymphatic Metastasis, Diabetes Mellitus, Type 2, Disease Progression, Hepatitis B, Acquired Immunodeficiency Syndrome, Carcinoma, Hepatocellular, Carcinoma, Squamous Cell, Celiac Disease, Colorectal Neoplasms, Coronary Disease, Lymphoma, Non-Hodgkin, Mouth Neoplasms, Multiple Sclerosis, Neoplasm Invasiveness, Thyroid Neoplasms, Hyperglycemia, Liver Neoplasms | Cytokine-cytokine receptor interaction, Chemokine signaling pathway,  Endocytosis, Axon guidance,  Leukocyte transendothelial migration,  Intestinal immune network for IgA production |
| CDKN1A  **GeneID:** 1026 | Cyclin-dependent kinase inhibitor 1A  **Chromosome:** 6; **Location:** 6p21.2 | 54 | 68 | Breast Neoplasm, Carcinoma, Squamous Cell, Ovarian Neoplasm, Prostatic Neoplasms, Stomach Neoplasm, Adenocarcinoma, Colorectal Neoplasms, Mouth Neoplasms, Lupus Erythematosus, Systemic Esophageal Neoplasms, Uterine Cervical Neoplasms, Urinary Bladder Neoplasms, Lung Neoplasms, Skin Neoplasms, Precancerous Conditions, DNA Damage, Melanoma, Head and Neck Neoplasms, Neoplasm Invasiveness, Neoplasm Recurrence, Local, Neoplasms, Glandular and Epithelial, Neuroma, Acoustic, Occupational Diseases , Multiple Endocrine Neoplasia Type 1, Myocardial Infarction, Nasopharyngeal Neoplasms, Helicobacter Infections, Intestinal Neoplasms, Laryngeal Neoplasms, Leiomyoma, Leukemia, Lymphocytic, Acute, L1, Leukemia, Pre-B-Cell, Meningioma, Lupus Nephritis, Lymphoma, Non-Hodgkin, Glaucoma, Open-Angle, Glioma, Disease Progression, Carcinoma, Carcinoma, Basal Cell, Atherosclerosis, Bowen's Disease, Brain Neoplasms, Carcinoma, Transitional Cell, Chromosome Aberrations, Chronic Disease, Prostatic Hyperplasia, Radiation Injuries, Skin Diseases, Pancreatic Neoplasms, Papillomavirus Infections, Pharyngeal Neoplasms, Uterine Neoplasms | ErbB signaling pathway  Cell cycle  p53 signaling pathway  Pathways in cancer  Glioma  Prostate cancer  Melanoma  Bladder cancer  Chronic myeloid leukemia |
| IRF1  **GeneID:** 3659 | interferon regulatory factor 1  **Chromosome:** 5; **Location:** 5q31.1 | 29 | 34 | Asthma, Arthritis, Juvenile Rheumatoid, Bronchiolitis, Viral Hypersensitivity, Respiratory Syncytial Virus Infections, Hepatitis C, Chronic, Multiple Sclerosis, Bronchial Hyperreactivity, Uterine Cervical Neoplasms, Papillomavirus Infections, Hypersensitivity, Subacute Sclerosing Panencephalitis, Thrombosis, Tuberculosis, Infant, Premature, Diseases, Lung Neoplasms, Malaria, Falciparum, Graft vs Host Disease, Graves Disease, Hepatitis B, Hepatitis C, Celiac Disease, Crohn Disease, Disease Progression, Disease Susceptibility, Acute Disease, Behcet Syndrome, Breast Neoplasms |  |
| TLR2  **GeneID:** 7097 | toll-like receptor 2  **Chromosome:** 4; **Location:** 4q32 | 120 | 136 | Asthma, Chronic Disease, Tuberculosis, Pulmonary, Periodontitis, Crohn Disease, Helicobacter Infections, Disease Progression, Acute Disease, Bacteremia, Pneumococcal Infections, Malaria Falciparum, Respiratory Syncytial Virus Infections, Premature Birth, Disease Susceptibility, Diabetes Mellitus, Type 1, Colitis, Ulcerative, HIV Infections, Hypersensitivity, Immediate, Inflammation, Lung Diseases, Mycobacterium Infections, Atypical, Hypersensitivity, Infection, Hepatitis C, Chronic, Cytomegalovirus Infections, Gastritis Duodenal Ulcer, Behcet Syndrome, Bronchiolitis, Viral Chagas Cardiomyopathy, Arthritis, Rheumatoid, Aspergillosis, Mycobacterium avium-intracellulare Infection, Recurrence Staphylococcal Infections, Urinary Tract Infections, Uterine Cervical Neoplasms, Vaginosis, Bacterial Stomach Neoplasms, Tuberculosis, Meningeal Tuberculosis, Pleural, Tuberculosis, Lymph Node, Systemic Inflammatory Response Syndrome, Virus Diseases, Waldenstrom Macroglobulinemia, Stevens-Johnson Syndrome, Prostatic Neoplasms, Pulmonary Disease, Chronic Obstructive, Pyelonephritis, Q Fever, Respiratory Tract Infections, Rheumatic Fever, Rheumatic Heart Disease, Rhinitis, Salmonella Infections, Sarcoidosis Sarcoidosis, Pulmonary Sepsis, Shock, Septic, Sinusitis, Skin Diseases, Spondylitis, Ankylosing, Meningococcal Infections, Metaplasia, Musculoskeletal Diseases, Pneumonia, Pneumonia, Pneumococcal, Pre-Eclampsia, Pregnancy Complications, Hematologic, Pregnancy Complications, Infectious, Aspergillosis, Allergic Bronchopulmonary, Atherosclerosis, Agricultural Workers' Diseases, Amyloidosis, Arthritis, Reactive Chlamydia Infections, Candidiasis, Carotid Artery Diseases, Bronchial Hyperreactivity, Bronchiectasis, Bacterial Infections, Elephantiasis, Filarial, Epidermal Necrolysis, Toxic Familial Mediterranean Fever, Fetal Diseases, Cystitis, Dental Plaque, Dermatitis, Atopic, Diabetes Complications, Colorectal Neoplasms, Connective Tissue Diseases, Coronary Disease, Coronary Restenosis, Herpes Genitalis, Hodgkin Disease, Hematologic Neoplasms, Gingival Hemorrhage, Gram-Negative Bacterial Infections, Infant, Premature, Diseases, Inflammatory Bowel Diseases, Leprosy, Lepromatous, Leukemia, Myeloid, Mycoses, Myocardial Infarction, Obesity, Otitis Media, Pancreatitis, Acute Necrotizing , Papillomavirus Infections, Peptic Ulcer, Periodontal Diseases, Lung Diseases, Fungal , Lung Neoplasms, Lupus Erythematosus, Systemic, Lyme Disease, Lymphoma, Lymphoma, Non-Hodgkin, Lymphoproliferative Disorders, Malaria | Toll-like receptor signaling pathway |
| CD14  **GeneID:** 929 | CD14 molecule  **Chromosome:** 5; **Location:** 5q31.1 | 166 | 253 | Asthma,Inflammation, Hypersensitivity, Immediate, Myocardial Infarction, Sepsis, Crohn Disease, Periodontitis, Colitis, Ulcerative, Chronic Disease, Helicobacter Infections, Disease Progression, Coronary Arteriosclerosis, Brain Ischemia, Bronchial Hyperreactivity, Burns Acute Disease, Cerebrovascular Accident, Stomach Neoplasms, Stroke, Carotid Artery Diseases, Chlamydia Infections, Chlamydophila Infections, Coronary Disease, Premature Birth, Recurrence, Respiratory Syncytial Virus Infections, Liver Diseases, Alcoholic, Hypersensitivity, Infection, Hepatitis C, Chronic Inflammatory Bowel Diseases, Liver Cirrhosis, Shock, Septic, Cardiovascular Diseases, Disease Susceptibility, Food Hypersensitivity , Arthritis, Rheumatoid, Alzheimer Disease, Atherosclerosis, Arteriosclerosis, Angina Pectoris, Adenocarcinoma, Alveolar Bone Loss, Bronchiolitis, Viral, Chronic Periodontitis, Cerebrovascular Disorders, Eczema, Critical Illness, Diabetes Mellitus, Type 1, Diabetes Mellitus, Type 2, Cystic Fibrosis, Colorectal Neoplasms, Communicable Diseases, Rhinitis, Allergic, Perennial, Sarcoidosis, Systemic Inflammatory Response Syndrome, Osteoporosis, Liver Cirrhosis, Alcoholic, Multiple Organ Failure, Intracranial Arteriosclerosis, Obesity , Pancreatitis, Pancreatitis, Gram-Negative Bacterial Infections, Hypertension, Kidney Failure, Chronic Spondylitis, Ankylosing , Tuberculosis, Pulmonary, Wounds and Injuries, Wounds, Nonpenetrating , Vaginosis, Bacterial Syndrome, Infant, Premature, Diseases, Guillain-Barre Syndrome, Herpesviridae Infections, Hodgkin Disease, Lung Diseases, Lung Neoplasms, Lymphoma, B-Cell, Marginal Zone, Lymphoma, Mucosa-Associated Lymphoid Tissue, Lymphoma, Non-Hodgkin, Migraine Disorders, Mucocutaneous Lymph Node Syndrome, Parkinson Disease, Periapical Periodontitis, Periodontal Attachment Loss, Periodontal Pocket, Occupational Diseases, Myocardial Ischemia, Kidney Diseases, Multiple Sclerosis, Musculoskeletal Diseases, Liver Diseases, Otitis Media, Respiratory Sounds, Proteinuria Pseudomonas Infections, Psoriasis, Peripheral Vascular Diseases, Pneumococcal Infections, Pneumonia, Bacterial Pouchitis, Pre-Eclampsia, Pregnancy Complications, Hematologic, Pregnancy Complications, Infectious, Thromboangiitis Obliterans, Thrombosis, Tooth Diseases, Toxoplasmosis, Trachoma, Tuberculosis, Tuberculosis, Miliary, Tuberculosis, Pleural, Schizophrenia, Rhinitis, Allergic, Seasonal, Skin Diseases, Spondylarthropathies, Severe Acute Respiratory Syndrome, Conjunctivitis, Connective Tissue Diseases, Constriction, Pathologic Coronary Restenosis, Coronary Stenosis, Cytomegalovirus Infections, Dermatitis, Atopic, Encephalitis, Enterocolitis, Necrotizing, Enterovirus Infections, Eosinophilia, Erythema Nodosum, Esophageal, Neoplasms, Fallopian Tube Diseases, Familial Mediterranean Fever, Farmer's Lung , Fetal Diseases, Fetal Membranes, Premature Rupture, Gastritis, Glomerulonephritis, Graft vs Host Disease, Hepatic Encephalopathy, Hepatitis B, Hepatitis B, Carotid Stenosis, Celiac Disease, Cholestasis, Brucellosis,Cadaver, Campylobacter Infections, Bronchiolitis, Aggressive Periodontitis, Agricultural Workers' Diseases, Airway Obstruction, Alcoholism, Amyloidosis, Anaphylaxis, Abortion, Habitual, Abscess, Angina, Appendicitis, Arthritis, Juvenile Rheumatoid, Arthritis, Reactive, Atrophy, Bacteremia, Biliary Atresia | MAPK signaling pathway, Toll-like receptor signaling pathway,  Hematopoietic cell lineage,  Regulation of actin cytoskeleton,  Pathogenic Escherichia coli infection – EHEC, Pathogenic Escherichia coli infection – EPEC, |
| IL18  **GeneID:** 3606 | interleukin 18 (interferon-gamma-inducing factor)  **Chromosome:** 11; **Location:** 11q22.2-q22.3 | 118 | 149 | Asthma, Diabetes Mellitus, Type 1, Inflammation, Cardiovascular Diseases, Coronary Disease, Arthritis, Rheumatoid, Crohn Disease, Colitis, Ulcerative Carcinoma, Squamous Cell, Hepatitis B, Chronic, Lupus Erythematosus, Systemic, Metabolic Syndrome X, Myocardial Infarction, Nasopharyngeal Neoplasms, Neoplasms, Disease Progression, Bronchiolitis, Viral, Alzheimer Disease, Respiratory Syncytial Virus Infections, Sarcoidosis, Obesity, Ovarian Neoplasms, Premature Birth, Stomach Neoplasms, Uterine Cervical Neoplasms, Uterine Neoplasms, Rhinitis, Allergic, Perennial, Rhinitis, Allergic, Seasonal , Arthritis, Arthritis, Juvenile Rheumatoid, Carcinoma, Hepatocellular, Autoimmune Diseases, Behcet Syndrome, Dermatitis, Atopic, Diabetes Complications, Diabetes Mellitus, Mucocutaneous Lymph Node Syndrome, Insulin Resistance, Hepatitis C, Chronic Helicobacter Infections, Graves Disease, Infection, Hodgkin Disease, Hypersensitivity, Immediate, Infant, Premature, Diseases, Hydatidiform Mole, Hypersensitivity, Inflammatory Bowel Diseases, Graves Ophthalmopathy, Head and Neck Neoplasms, Gingivitis, Graft vs Host Disease, Hemophilia A, Hepatitis B, HIV Infections, Intracranial Arteriosclerosis, Latex Hypersensitivity, Leiomyoma, Lichen Planus, Liver Neoplasms, Lung Neoplasms, Lupus Nephritis, Lymphoma, Lymphoma, Non-Hodgkin Lymphoproliferative Disorders, Macular Degeneration, Musculoskeletal Diseases, Metaplasia, Mouth Neoplasms, Glandular and Epithelial, Diabetes Mellitus, Type 2, Duodenal Ulcer, Dyspepsia, Endotoxemia, Epstein-Barr Virus Infections, Esophageal Neoplasms, Fetal Diseases, Colorectal Neoplasms, Connective Tissue Diseases, Death, Sudden, Cardiac, Celiac Disease, Choriocarcinoma, Choroidal Neovascularization, Chronic Periodontitis, Cognition Disorders, Brain Ischemia, Breast Neoplasms, Bronchial Hyperreactivity, Carcinoma, Atherosclerosis, Abortion, Habitual, Abortion, Spontaneous, Adenocarcinoma, Aggressive Periodontitis, Uveitis, Vasculitis, Waldenstrom Macroglobulinemia, Wounds and Injuries, Stomach Ulcer, Stroke, Substance Abuse, Intravenous, Thyroid Diseases, Tuberculosis, Pulmonary Prostatic Neoplasms, Proteinuria, Recurrence, Periodontitis, Pneumoconiosis, Postoperative Complications, Precancerous Conditions, Pregnancy Complications, Hematologic, Osteoporosis,Retinal Vein Occlusion, Sarcoidosis, Pulmonary, Schistosomiasis mansoni, Schizophrenia, Skin Diseases, Still's Disease, Adult-Onset | Cytokine-cytokine receptor interaction,  NOD-like receptor signaling pathway, Cytosolic DNA-sensing pathway |
| LEP  **GeneID:** 3952 | Leptin  **Chromosome:** 7; **Location:** 7q31.3 | 55 | 104 | Obesity, Hypertension, Weight Gain, Schizophrenia, Obesity, Morbid, Diabetes Mellitus, Type 2, Pre-Eclampsia, Lymphoma, Non-Hodgkin, Insulin Resistance, Breast Neoplasms, Prostatic Neoplasms, Pregnancy in Diabetics, Behcet Syndrome, Birth Weight , Disease Progression , Chronic Disease, Colonic Neoplasms, Diabetes Mellitus, Type 1, Lung Neoplasms, Carcinoma, Squamous Cell, Cardiovascular Diseases, HELLP Syndrome, Hepatitis, Toxic, Kidney Failure, Chronic, Mental Disorders, Metabolic Syndrome X, Microsatellite Instability, Mouth Neoplasms, Neovascularization, Pathologic, Pregnancy Complications, Cardiovascular, Osteoporosis, Postmenopausal, Colorectal Neoplasms, Coronary Restenosis, Depressive Disorder , Diabetes Mellitus, Diseases in Twins, Dyslipidemias, Endometrial Neoplasms, Esophageal Neoplasms, Body Weight, Carcinoma, Carcinoma, Non-Small-Cell Lung, Adenocarcinoma, Amyotrophic Lateral Sclerosis, Anoxia, Asthma, Atherosclerosis, Attention Deficit Disorder with Hyperactivity, Autistic Disorder , Psoriasis, Puberty Delayed , Weight Loss, Sleep Apnea, Uterine Neoplasms | Neuroactive ligand-receptor interaction, Cytokine-cytokine receptor interaction, Adipocytokine signaling pathway,  Jak-STAT signaling pathway, |
| LEPR  **GeneID:** 3953 | leptin receptor  **Chromosome:** 1; **Location:** 1p31 | 79 | 137 | Obesity, Hypertension, Weight Gain, Schizophrenia, Obesity, Morbid, Diabetes Mellitus, Type 2, Pre-Eclampsia, Lymphoma, Non-Hodgkin, Insulin Resistance, Breast Neoplasms, Prostatic Neoplasms, Pregnancy in Diabetics, Behcet Syndrome, Birth Weight, Disease Progression, Chronic Disease, Colonic Neoplasms, Diabetes Mellitus, Type 1, Lung Neoplasms, Carcinoma, Squamous Cell, Cardiovascular Diseases, HELLP Syndrome, Hepatitis, Kidney Failure, Mental Disorders, Metabolic Syndrome X, Microsatellite Instability, Mouth Neoplasms, Neovascularization, Pathologic Pregnancy Complications, Cardiovascular, Osteoporosis, Postmenopausal, Colorectal Neoplasms, Coronary Restenosis, Depressive Disorder , Diabetes Mellitus, Diseases in Twins, Dyslipidemias, Endometrial Neoplasms, Esophageal Neoplasms, Body Weight, Carcinoma, Non-Small-Cell Lung , Adenocarcinoma, Amyotrophic Lateral Sclerosis, Anoxia, Asthma, Atherosclerosis, Attention Deficit Disorder with Hyperactivity, Autistic Disorder, Psoriasis, Puberty, Delayed, Weight Loss, Sleep Apnea, Uterine Neoplasms | Neuroactive ligand-receptor interaction, Cytokine-cytokine receptor interaction, Adipocytokine signaling pathway,  Jak-STAT signaling pathway |
| IGF1R  **GeneID:** 3480 | insulin-like growth factor 1 receptor  **Chromosome:** 15; **Location:** 15q26.3 | 51 | 45 | Diabetes Mellitus, Type 2, Insulin Resistance, Obesity, Multiple Myeloma, Retinopathy of Prematurity, Hypertension, Metabolic Syndrome X, Glucose Intolerance, Alzheimer Disease, Body Weight, Breast Neoplasms, Carcinoma, Squamous Cell, Cardiovascular Diseases, Colonic Neoplasms, Dementia, Dementia, Diabetes Complications, Diabetes Mellitus, Bone Diseases, Brain Ischemia, Brain Neoplasms, Anemia, Sickle Cell, Bacteremia, Barrett Esophagus, Birth Weight, Growth Disorders, Head and Neck Neoplasms, Disease Progression, Esophageal Neoplasms, Fetal Growth Retardation, Gastroesophageal Reflux, Microsatellite Instability, Adenocarcinoma, Intracranial Arteriosclerosis, Lung Neoplasms, Lymphoma, Non-Hodgkins, Schizophrenia, Spinal Diseases, Stomach Neoplasms, Stroke, Testicular Neoplasms , Neoplasms, Germ Cell and Embryonal,, Neoplasms, Glandular and Epithelial Neoplasms, Second Primary, Osteoporosis, Ovarian Neoplasms, Polycystic Ovary Syndrome | Oocyte meiosis, Endocytosis,  Focal adhesion,  Adherens junction,  Long-term depression, Progesterone-mediated oocyte maturation, Pathways in cancer, Colorectal cancer,  Glioma,  Prostate cancer,  Melanoma |
| REN  **GeneID:** 5972 | Rennin  **Chromosome:** 1; **Location:** 1q32 | 21 | 32 | Hypertension, Cardiovascular Diseases 2, Alzheimer Disease, Diabetes Mellitus, Type 2, Diabetic Nephropathies, Emergencies, Diabetic Angiopathies, Brain Ischemia, Carotid Stenosis, Cerebrovascular Accident, Diabetes Complications, Diabetes Mellitus, Glaucoma, Open-Angle, Hyperaldosteronism, Hyperlipidemias, Metabolic Syndrome X, Neoplasms, Osteoporosis, Pre-Eclampsia, Renal Insufficiency, Chronic Tobacco Use Disorder | Renin-angiotensin system |
| AKT1  **GeneID:** 207 | v-akt murine thymoma viral oncogene homolog 1  **Chromosome:** 14; **Location:** 14q32.32 | 34 | 40 | Schizophrenia, Amphetamine-Related Disorders, Bipolar Disorder, Disease Progression, Neoplasm Metastasis, Neoplasms, Neuropsychological Tests, Osteoporosis, Parkinson Disease, Precursor T-Cell Lymphoblastic Leukemia-Lymphoma, Psychiatric Status Rating Scales, Psychotic Disorders, Retinal Neoplasms, Retinoblastoma, Dominance, Cerebral, Drug Toxicity, Dyskinesia, Drug-Induced Edema, Endometrial Neoplasms, Esophageal Neoplasms, Carcinoma, Papillary, Follicular Cardiovascular Diseases, Cognition Colorectal Neoplasms, Diabetes Complications, Diabetes Mellitus, Type 2, Basal Ganglia Diseases 1, Alzheimer Disease, Lung Neoplasms, Memory, Metabolic Syndrome X, Space Perception, Thyroid Neoplasms | Insulin signaling pathway, Adipocytokine signaling pathway,  MAPK signaling pathway, Jak-STAT signaling pathway,  Focal adhesion,  Prostate cancer,  Small cell lung cancer, Toll-like receptor signaling pathway,  Apoptosis,  T cell receptor signaling pathway,  Fc epsilon RI signaling pathway,  Colorectal cancer, Pancreatic cancer,  Glioma,  Melanoma,  Non-small cell lung cancer,  Renal cell carcinoma, VEGF signaling pathway, Endometrial cancer, Chronic myeloid leukemia, mTOR signaling pathway, ErbB signaling pathway, Tight junction,  Acute myeloid leukemia, B cell receptor signaling pathway,  Chemokine signaling pathway,  Fc gamma R-mediated phagocytosis, Neurotrophin signaling pathway,  Progesterone-mediated oocyte maturation, Pathways in cancer, |
| VCAM1  **GeneID:** 7412 | vascular cell adhesion molecule 1  **Chromosome:** 1; **Location:** 1p32-p31 | 42 | 36 | Inflammation, Brain Ischemia, Anemia, Sickle Cell, Cerebrovascular Accident, Respiratory Syncytial Virus Infections, Stroke, Asthma, Bronchiolitis, Viral, Cardiovascular Diseases, Myocardial Infarction, Hypertension, Osteoporosis, Alzheimer Disease, Infant, Premature, Diseases, Neoplasms, Obesity, Occupational Diseases, Lung Neoplasms, Lymphedema, Lymphoma, Large B-Cell, Diffuse Lymphoma, Non-Hodgkin, Lymphoproliferative Disorders Metabolic Syndrome X, Migraine Disorders , Multiple Sclerosis, Carotid Stenosis, Bronchial Hyperreactivity, Bronchiolitis, Atherosclerosis, Biliary Tract Neoplasms, Arthritis, Rheumatoid, Cerebrovascular Disorders, Diabetes Complications, Diabetes Mellitus, Type 2, Erythema Nodosum, Heart Diseases, Hematologic Diseases, Hodgkin Disease, Hypersensitivity, Immediate, Thrombophilia, Waldenstrom Macroglobulinemia, Sarcoidosis 1 | Cell adhesion molecules (CAMs),  Leukocyte transendothelial migration, |
| IKBKG  **GeneID:** 8517 | inhibitor of kappa light polypeptide gene enhancer in B-cells, kinase gamma  **Chromosome:** X; **Location:** Xq28 | 2 | 2 | Atherosclerosis,  Calcinosis | MAPK signaling pathway, Chemokine signaling pathway, Apoptosis, Toll-like receptor signaling pathway, NOD-like receptor signaling pathway, RIG-I-like receptor signaling pathway, Cytosolic DNA-sensing pathway, T cell receptor signaling pathway, B cell receptor signaling pathway, Adipocytokine signaling pathway, Epithelial cell signaling in Helicobacter pylori infection, Pathways in cancer, Pancreatic cancer, Prostate cancer, Chronic myeloid leukemia, Acute myeloid leukemia, Small cell lung cancer, Primary immunodeficiency |
| ERK  **GeneID:** 2080 | elk-related tyrosine kinase | 7 | 4 | Colorectal Neoplasms, Prostatic Neoplasms, Adenomatous Polyposis Coli, Colorectal Neoplasms, Hereditary Nonpolyposis, Intestinal Polyposis, Parkinson Disease, Precancerous Conditions 1 | Axon guidance |
| WNK1  **GeneID:** 65125 | WNK lysine deficient protein kinase 1  **Chromosome:** 12; **Location:** 12p13.3 | 1 | 6 | Hypertension 2 |  |
| SREBF1  **GeneID:** 6720 | sterol regulatory element binding transcription factor 1  **Chromosome:** 17; **Location:** 17p11.2 | 24 | 28 | Diabetes Mellitus, Type 2, Hypercholesterolemia, Insulin Resistance, Alzheimer Disease, Metabolic Syndrome X, Cardiovascular Diseases, Coronary Arteriosclerosis, Coronary Disease, Diabetes Complications, Diabetes Mellitus, Diabetes Mellitus, Type 1, Disease Progression, Drug Toxicity, Edema, Femur Head Necrosis, Neoplasms, Obesity, Osteoporosis, Renal Insufficiency, Chronic, Schizophrenia, Weight Gain, Arteriosclerosis, Hyperlipidemias, Kidney Failure, Chronic | Insulin signaling pathway, |
| MAPK1  **GeneID:** 5594 | mitogen-activated protein kinase 1  **Chromosome:** 22; **Location:** 22q11.21 | 2 | 4 | Asthma, Thyroid Neoplasms | MAPK signaling pathway, ErbB signaling pathway, Chemokine signaling pathway, Oocyte meiosis, mTOR signaling pathway, Vascular smooth muscle contraction, Dorso-ventral axis formation, TGF-beta signaling pathway, Axon guidance, VEGF signaling pathway, Focal adhesion, Adherens junction, Gap junction, Toll-like receptor signaling pathway, NOD-like receptor signaling pathway, Natural killer cell mediated cytotoxicity, T cell receptor signaling pathway, B cell receptor signaling pathway, Fc epsilon RI signaling pathway, Fc gamma R-mediated phagocytosis,Long-term potentiation, Neurotrophin signaling pathway, Long-term depression, Regulation of actin cytoskeleton, Insulin signaling pathway, GnRH signaling pathway, Progesterone-mediated oocyte maturation, Melanogenesis, Type II diabetes mellitus, Alzheimer's disease, Prion diseases, Pathways in cancer, Colorectal cancer, Renal cell carcinoma, Pancreatic cancer, Endometrial cancer, Glioma, Prostate cancer, Thyroid cancer, Melanoma, Bladder cancer, Chronic myeloid leukemia, Acute myeloid leukemia, Non-small cell lung cancer |
| EGFR  **GeneID:** 1956 | epidermal growth factor receptor  **Chromosome:** 7; **Location:** 7p12 | 69 | 261 | Lung Neoplasms, Carcinoma, Non-Small-Cell Lung, Adenocarcinoma, Carcinoma, Squamous Cell, Neoplasm Recurrence, Local, Disease Progression, Colorectal Neoplasms, Breast Neoplasms, Adenocarcinoma, Bronchiolo-Alveolar Carcinoma, Large Cell, Head and Neck Neoplasms, Brain Neoplasms, Diarrhea, Esophageal Neoplasms, Exanthema, Glioblastoma, Lymphatic Metastasis, Neoplasm Metastasis, Rectal Neoplasms, Pancreatic Neoplasms, Astrocytoma, Carcinoma, Hepatocellular, Carcinoma, Adenosquamous, Ovarian Neoplasms, Polycystic Kidney, Autosomal Dominant, Stomach Neoplasms, Urinary Bladder Neoplasms, Uterine Cervical Neoplasms, Colonic Neoplasms, Nasopharyngeal Neoplasms Neoplasms, Glandular and Epithelial, Neoplasms, Squamous Cell, Osteosarcoma | MAPK signaling pathway, ErbB signaling pathway, Calcium signaling pathway, Cytokine-cytokine receptor interaction, Endocytosis, Dorso-ventral axis formation, Focal adhesion, Adherens junction, Gap junction, Regulation of actin cytoskeleton, GnRH signaling pathway, Epithelial cell signaling in Helicobacter pylori infection, Pathways in cancer, Colorectal cancer, Pancreatic cancer, Endometrial cancer, Glioma, Prostate cancer, Melanoma, Bladder cancer, Non-small cell lung cancer |
| STAT5B  **GeneID:** 6777 | signal transducer and activator of transcription 5B  **Chromosome:** 17; **Location:** 17q11.2 | 4 | 4 | Breast Neoplasms, Cleft Lip, Cleft Palate, Tooth Abnormalities | ErbB signaling pathway, Chemokine signaling pathway, Jak-STAT signaling pathway, Pathways in cancer, Chronic myeloid leukemia, Acute myeloid leukemia |
| EP300  **GeneID:** 2033 | E1A binding protein p300  **Chromosome:** 22; **Location:** 22q13.2 | 6 | 8 | Breast Neoplasms, Ductus Arteriosus, Patent, Infant, Premature, Diseases, Neoplasms, Glandular and Epithelial, Ovarian Neoplasms, Thyroid Neoplasms | Cell cycle, Wnt signaling pathway, Notch signaling pathway, TGF-beta signaling pathway, Adherens junction, Jak-STAT signaling pathway, Long-term potentiation,Melanogenesis, Huntington's disease, Pathways in cancer, Renal cell carcinoma, Prostate cancer |
| HSP90AA1  **GeneID:** 3320 | heat shock protein 90kDa alpha (cytosolic), class A member 1  **Chromosome:** 14; **Location:** 14q32.33 | 1 | 2 | Asthma | Antigen processing and presentation,NOD-like receptor signaling pathway, Progesterone-mediated oocyte maturation, Pathways in cancer Prostate cancer |
| MAPK3  **GeneID:** 5595 | mitogen-activated protein kinase 3  **Chromosome:** 16; **Location:** 16p11.2 | 2 | 5 | Asthma, Autistic Disorder | MAPK signaling pathway, ErbB signaling pathway, Chemokine signaling pathway, Oocyte meiosis, mTOR signaling pathway, Vascular smooth muscle contraction ,Dorso-ventral axis formation, TGF-beta signaling pathway, Axon guidance, VEGF signaling pathway, Focal adhesion, Adherens junction, Gap junction, Toll-like receptor signaling pathway, NOD-like receptor signaling pathway, Natural killer cell mediated cytotoxicity, T cell receptor signaling pathway, B cell receptor signaling pathway, Fc epsilon RI signaling pathway, Fc gamma R-mediated phagocytosis,Long-term potentiation, Neurotrophin signaling pathway, Long-term depression, Regulation of actin cytoskeleton, Insulin signaling pathway, GnRH signaling pathway, Progesterone-mediated oocyte maturation, Melanogenesis,Type II diabetes mellitus, Alzheimer's disease, Prion diseases, Pathways in cancer, Colorectal cancer, Renal cell carcinoma. Pancreatic cancer, Endometrial cancer, Glioma, Prostate cancer, Thyroid cancer, Melanoma, Bladder cancer, Chronic myeloid leukemia, Acute myeloid leukemia, Non-small cell lung cancer |
| MAPK8  **GeneID:** 5599 | mitogen-activated protein kinase 8  **Chromosome:** 10; **Location:** 10q11.22 | 2 | 3 | Breast Neoplasms, Diabetes Mellitus, Type 2 | Insulin signaling pathway, Adipocytokine signaling pathway,  MAPK signaling pathway, Type II diabetes mellitus, Focal adhesion,  Toll-like receptor signaling pathway,  Fc epsilon RI signaling pathway,  Colorectal cancer, Pancreatic cancer,  Wnt signaling pathway, ErbB signaling pathway, GnRH signaling pathway, Epithelial cell signaling in Helicobacter pylori infection,  NOD-like receptor signaling pathway RIG-I-like receptor signaling pathway Neurotrophin signaling pathway Progesterone-mediated oocyte maturation Adipocytokine signaling pathway Pathways in cancer |
| IRS1  **GeneID:** 3667 | insulin receptor substrate 1  **Chromosome:** 2; **Location:** 2q36 | 52 | 99 | Diabetes Mellitus, Type 2, Insulin Resistance, Obesity, Polycystic Ovary Syndrome, Breast Neoplasms, Diabetes Mellitus, Hyperandrogenism, Hypertension, Glucose Intolerance, Metabolic Syndrome X, Colonic Neoplasms, Colorectal Neoplasms, Hyperinsulinism, Puberty, Precocious, Prostatic Neoplasms, Coronary Disease, Alzheimer Disease, Birth Weight, Body Weight, Diabetes Mellitus, Type 1, Diabetes, Gestational, Glucose Metabolism Disorders | Insulin signaling pathway, Adipocytokine signaling pathway,  Type II diabetes mellitus, Neurotrophin signaling pathway |
| RAC1  **GeneID:** 5879 | ras-related C3 botulinum toxin substrate 1  **Chromosome:** 7; **Location:** 7p22 | 5 | 7 | Lung Neoplasms, Lymphoma, Non-Hodgkin, Mental Retardation, Occupational Diseases, Brain Neoplasms | MAPK signaling pathway, Chemokine signaling pathway,  Wnt signaling pathway, Axon guidance,  VEGF signaling pathway, Focal adhesion,  Adherens junction,  Toll-like receptor signaling pathway,  Natural killer cell mediated cytotoxicity,  B cell receptor signaling pathway,  Fc epsilon RI signaling pathway,  Fc gamma R-mediated phagocytosis,  Leukocyte transendothelial migration,  Neurotrophin signaling pathway,  Regulation of actin cytoskeleton,  Amyotrophic lateral sclerosis (ALS),  Epithelial cell signaling in Helicobacter pylori infection,  Pathways in cancer, Colorectal cancer,  Renal cell carcinoma, Pancreatic cancer,  Viral myocarditis |
| CDKN2A  **GeneID:** 1029 | cyclin-dependent kinase inhibitor 2A  **Chromosome:** 9; **Location:** 9p21 | 89 | 166 | Diabetes Mellitus, Type 2,Melanoma, Skin Neoplasms, Breast Neoplasms, Pancreatic Neoplasms, Carcinoma, Squamous Cell, Adenocarcinoma, Ovarian Neoplasms, Esophageal Neoplasms, Neoplasms, Multiple Primary, Myocardial Infarction, Disease Progression, Colorectal Neoplasms, Coronary Disease, Lung Neoplasms, Head and Neck Neoplasms, Glucose Intolerance, Insulin Resistance, Barrett Esophagus, Birth Weight, Stroke, Urinary Bladder Neoplasms, Uveal Neoplasms, Stomach Neoplasms, Precancerous Conditions, Brain Ischemia, Brain Neoplasms, Cystadenocarcinoma, Coronary Arteriosclerosis, Carcinoma Diabetes, Gestational Diabetes Mellitus 2, Diabetes Mellitus, Type 1, Neoplasms, Second Primary, Neoplasm Invasiveness, Neoplasms, Meningioma, Mouth Neoplasms, Glioma, Hyperglycemia, Hyperlipoproteinemia Type II,Hypertension | Pancreatic cancer,  Glioma,  Melanoma,  Non-small cell lung cancer,  Chronic myeloid leukemia, p53 signaling pathway, Cell cycle,  Bladder cancer,  Pathways in cancer |
| CDKN2B  **GeneID:** 1030 | cyclin-dependent kinase inhibitor 2B  **Chromosome:** 9; **Location:** 9p21 | 42 | 87 | Diabetes Mellitus, Type 2, Coronary Disease, Insulin Resistance, Melanoma, Myocardial Infarction, Ovarian Neoplasms, Glucose Intolerance, Breast Neoplasms, Birth Weight, Colorectal Neoplasms, Coronary Arteriosclerosis, Diabetes Mellitus, Diabetes Mellitus, Type 1, Diabetes, Gestational, Glioma, Hyperglycemia, Hyperlipoproteinemia Type II, Hypertension, Skin Neoplasms, Stroke, Obesity | Small cell lung cancer, TGF-beta signaling pathway,  Cell cycle, |
| NOS3  **GeneID:** 4846 | nitric oxide synthase 3  **Chromosome:** 7; **Location:** 7q36 | 262 | 711 | Hypertension, Diabetes Mellitus, Type 2, Coronary Arteriosclerosis, Cardiovascular Diseases, Coronary Disease, Myocardial Infarction, Kidney Failure, Pre-Eclampsia, Disease Progression, Diabetic Nephropathies, Alzheimer Disease, Asthma, Stroke, Breast Neoplasms, Diabetic Retinopathy, Inflammation, Brain Ischemia, Atherosclerosis, Diabetes Mellitus, Type 1, Hypertrophy, Left Ventricular, Acute Disease, Carotid Artery Diseases, Cerebrovascular Accident, Insulin Resistance, Diabetic Angiopathies, Coronary Vasospasm, Subarachnoid Hemorrhage, Thrombosis, Prostatic Neoplasms, Recurrence, Metabolic Syndrome X , Myocardial Ischemia, Abortion, Habitual, Arteriosclerosis, Behcet Syndrome, Diabetes Complications, Intracranial Aneurysm, Hyperhomocysteinemia, Obesity, Polycystic Kidney, Autosomal Dominant, Thrombophilia, Vasospasm, Intracranial, Syndrome, Kidney Diseases, Impotence, Diabetes Mellitus, Disease Susceptibility, Heart Defects, Congenital, Arthritis, Rheumatoid, Atrial Fibrillation, Albuminuria, Anemia, Sickle Cell, Chronic Disease, Colorectal Neoplasms, Coronary Restenosis, Coronary Stenosis, Chest Pain, Cerebral Palsy, Aneurysm, Ruptured Altitude Sickness, Constriction, Pathologic Vascular Diseases, Osteoporosis, Pulmonary Edema, Neovascularization, Migraine Disorders, Neoplasms, Microvascular Angina, Lymphoma, Non-Hodgkin Lung Neoplasms, Lupus Erythematosus, Systemic, Scleroderma, Premature Birth, Pulmonary Disease, Chronic Obstructive, Spinal Dysraphism, Heart Diseases, Heart Failure, Congestive, Glomerulonephritis, Hypercholesterolemia, Hyperemia, Angina, Unstable, Anoxia, Abruptio Placentae, Arterial Occlusive Diseases, Carcinoma, Carcinoma, Intraductal, Noninfiltrating, Cardiomegaly, Coronary Thrombosis, Cystic Fibrosis, Coronary Artery Disease, Aortic Aneurysm, Angina Pectoris Brain Infarction, Birth Weight, Graft Occlusion, Vascular, HIV Infections, Hypertension, Hyperlipoproteinemia Type II, Ischemia, Hypertriglyceridemia, Glomerulonephritis, IGA, Glaucoma, Open-Angle, Fabry Disease, Femur Head Necrosis, Endometriosis, Skin Diseases, Temporal Arteritis, Thromboangiitis Obliterans, Pregnancy Complications, Cardiovascular, Peripheral Vascular Diseases, Postoperative Complications, Osteoporosis, Postmenopausal, Parkinson Disease, Retinopathy of Prematurity, Renal Artery Obstruction, Lupus Nephritis, Lewy Body Disease, Lead Poisoning, Neoplasm Metastasis, Migraine with Aura | Calcium signaling pathway,  Small cell lung cancer, VEGF signaling pathway, Long-term depression, Arginine and proline metabolism,  Metabolic pathways, Alzheimer's disease, Amyotrophic lateral sclerosis (ALS),  Pathways in cancer |
| KCNJ11  **GeneID:** 3767 | potassium inwardly-rectifying channel, subfamily J, member 11  **Chromosome:** 11; **Location:** 11p15.1 | 29 | 88 | Diabetes Mellitus, Type 2, Insulin Resistance, Diabetes Mellitus, Type 1, Glucose Intolerance, Diabetes Mellitus, Hypertension, Diabetes, Gestational, Obesity, Persistent Hyperinsulinemia Hypoglycemia of Infancy, Polycystic Ovary Syndrome, Metabolic Syndrome X, Myocardial Infarction, Birth Weight, Coronary Disease, Hypoglycemia, Hyperglycemia, Disease Progression | Type II diabetes mellitus |
| TCF7L2  **GeneID:** 6934 | transcription factor 7-like 2  **Chromosome:** 10; **Location:** 10q25.3 | 53 | 160 | Diabetes Mellitus, Type 2, Insulin Resistance, Obesity, Diabetes Mellitus, Diabetes, Gestational, Glucose Intolerance, Diabetic Nephropathies, Atherosclerosis, Diabetes Mellitus, Type 1, Colonic Neoplasms, Prostatic Neoplasms, Metabolic Syndrome X, Hypertension, Polycystic Ovary Syndrome, Diabetic Angiopathies, Alzheimer Disease, Breast Neoplasms, Disease Progression, Fatty Liver, Hyperglycemia, Hyperinsulinism | Prostate cancer,  Colorectal cancer,  Wnt signaling pathway, Endometrial cancer, Adherens junction,  Acute myeloid leukemia, Melanogenesis,  Basal cell carcinoma, Thyroid cancer |
| HHEX  **GeneID:** 3087 | hematopoietically expressed homeobox  **Chromosome:** 10; **Location:** 10q23.33 | 16 | 64 | Diabetes Mellitus, Type 2,Glucose Intolerance, Insulin Resistance, Diabetes Mellitus, Type 1, Birth Weight, Alzheimer Disease, Hyperglycemia, Diabetes, Gestational, Obesity | Maturity onset diabetes of the young |
| SLC30A8 |  | 19 | 68 | Diabetes Mellitus, Type 2, Insulin Resistance, Diabetes Mellitus, Type 1, Obesity, Birth Weight, Diabetes Mellitus, Glucose Intolerance, Hyperglycemia, Diabetes, Gestational |  |
